# E3 Ubiquitin ligase ZNRF4 negatively regulates NOD2 signalling and induces tolerance to MDP

**DOI:** 10.1038/ncomms15865

**Published:** 2017-06-28

**Authors:** Pradeep Bist, Wan Shoo Cheong, Aylwin Ng, Neha Dikshit, Bae-Hoon Kim, Niyas Kudukkil Pulloor, Hanif Javanmard Khameneh, Matija Hedl, Avinash R. Shenoy, Vanniarajan Balamuralidhar, Najib Bin Abdul Malik, Michelle Hong, Albert Neutzner, Keh-Chuang Chin, Koichi S. Kobayashi, Antonio Bertoletti, Alessandra Mortellaro, Clara Abraham, John D. MacMicking, Ramnik J. Xavier, Bindu Sukumaran

**Affiliations:** 1 Program in Emerging Infectious Diseases, Duke-NUS Medical School, Singapore 169857, Singapore; 2 Gastrointestinal Unit, Center for Computational and Integrative Biology, Massachusetts General Hospital, Harvard Medical School, Boston, Massachusetts 02114, USA; 3 Broad Institute of Harvard and MIT, Cambridge, Massachusetts 02142, USA; 4 HHMI, Yale Systems Biology Institute, Departments of Microbial Pathogenesis and Immunobiology, Yale University School of Medicine, New Haven, Connecticut 065207, USA; 5 Singapore Immunology Network (SIgN), Agency for Science, Technology and Research (A*STAR), Singapore 138648, Singapore; 6 Department of Internal Medicine, Yale University School of Medicine, New Haven, Connecticut 06520, USA; 7 Medical Research Council Centre for Molecular Bacteriology & Infection, Armstrong Rd, Imperial College, London SW7 2AZ, UK; 8 Department of Biomedicine, University Hospital Basel, Basel 4031, Switzerland; 9 Department of Physiology, Yong Loo Lin School of Medicine, Singapore 117593, Singapore; 10 Institute of Molecular and Cell Biology, A*STAR, Singapore 138673, Singapore; 11 Department of Microbial Pathogenesis and Immunology, Texas A&M Health Science Centre, College Station, Texas 77843-1114, USA

## Abstract

Optimal regulation of the innate immune receptor nucleotide-binding oligomerization domain-containing protein 2 (NOD2) is essential for controlling bacterial infections and inflammatory disorders. Chronic NOD2 stimulation induces non-responsiveness to restimulation, termed NOD2-induced tolerance. Although the levels of the NOD2 adaptor, RIP2, are reported to regulate both acute and chronic NOD2 signalling, how RIP2 levels are modulated is unclear. Here we show that ZNRF4 induces K48-linked ubiquitination of RIP2 and promotes RIP2 degradation. A fraction of RIP2 localizes to the endoplasmic reticulum (ER), where it interacts with ZNRF4 under either unstimulated and muramyl dipeptide-stimulated conditions. *Znrf4* knockdown monocytes have sustained nuclear factor kappa-light-chain-enhancer of activated B cells (NF-κB) activation, and *Znrf4* knockdown mice have reduced NOD2-induced tolerance and more effective control of *Listeria monocytogenes* infection. Our results thus demonstrate E3-ubiquitin ligase ZNRF4-mediated RIP2 degradation as a negative regulatory mechanism of NOD2-induced NF-κB, cytokine and anti-bacterial responses *in vitro* and *in vivo*, and identify a ZNRF4-RIP2 axis of fine-tuning NOD2 signalling to promote protective host immunity.

## Abstract (web summary)

Prolonged NOD2 ligand engagement induces tolerance and attenuated NOD2 signalling, but the molecular mechanisms leading to this tolerance induction are unclear. Here the authors show that the degradation of a NOD2 adaptor, RIP2, by the E3 ligase ZNRF4 is essential for the down-regulation of NOD2 signalling.

Nucleotide-binding oligomerization domain-containing protein 1 (NOD1) and nucleotide-binding oligomerization domain-containing protein 2 (NOD2) are critical intracellular pattern recognition receptors (PRRs) needed to control bacterial pathogens. NOD2 recognizes muramyl dipeptide (MDP), a peptidoglycan component from both Gram-positive and Gram-negative bacteria^1^,^2^, and triggers immune responses through the activation of nuclear factor kappa-light-chain-enhancer of activated B cells (NF-κB), mitogen-activated protein kinases (MAPKs), autophagy and the inflammasome^3^. Moreover, polymorphisms in NOD2 and aberrant NOD2 signalling are identified as major susceptibility factors of several inflammatory disorders such as Crohn’s disease, Blau syndrome, early onset sarcoidosis, autoimmune disease, allergy and asthma^4^. Effective control of NOD2 signalling is thus critical to reduce excessive inflammation during both infection and other immune-mediated diseases.

Binding of MDP to its receptor NOD2 leads to the association of NOD2 with the adaptor protein RIP2, which subsequently interacts with TAK1, and differentially recruits NEMO and MKKs to activate NF-κB and MAPKs, respectively^5^,^6^,^7^,^8^,^9^. Previous studies have identified several positive regulators of NOD2 signalling^10^,^11^,^12^,^13^,^14^. Despite the progress that has been made^15^,^16^, several important questions remain unanswered. Particularly, the negative feedback mechanisms that attenuate the NOD2 pathway during both acute and chronic stimulation have yet to be fully defined. Similar to the endotoxin shock induced by Gram-negative bacteria, Gram-positive bacterial pathogens (that lack lipopolysaccharide) also cause septic shock via ligands such as MDP. To counter septic shock, mammalian cells have evolved mechanisms in which prolonged stimulation with MDP induces non-responsiveness to further MDP stimulation (termed NOD2-induced tolerance)^17^,^18^,^19^. Although lipopolysaccharide (LPS) tolerance mechanisms are well known, the mechanisms governing tolerance to MDP and Gram-positive bacteria are incompletely understood.

How the cell tightly maintains the homeostasis level of RIP2 is also unknown. Previous studies have either proposed^20^ or identified^19^ ligand-stimulation-dependent degradation of RIP2 and NOD2 as potential mechanisms to attenuate NOD2 signalling. Although some proteins were reported to negatively regulate NOD2 signalling by modulating the level of NOD2 (refs 21, 22, 23, 24), very little information is available on the regulation of RIP2 degradation in NOD2 signalling attenuation. ITCH, caspase-12 and MEKK4 were reported to negatively regulate NOD2-induced NF-κB activation by competitively binding to RIP2 (refs 20, 25, 26). Degradation of PRR adaptors through K48 ubiquitination has been demonstrated as an important mechanism for attenuating innate immune response. Although positive regulation of NOD2 signalling by K63- or M1-linked ubiquitination of RIP2 by multiple E3-ubiquitin ligases, including cIAP1/2 (ref. 11), XIAP^12^,^16^,^27^ and Pellino3 (ref. 14), is well known, whether ubiquitination-dependent degradation of RIP2 has any function in attenuating NOD2 signalling is unclear.

Here we perform genome-wide RNA-interference (RNAi) screening in human embryonic kidney 293T (HEK293T) cells to identify genes and related mechanisms responsible for the negative regulation of NOD2-mediated NF-κB activation. Our data identify the E3-ubiquitin ligase ZNRF4 as a negative regulator of NOD2-mediated anti-bacterial and inflammatory responses during both acute and chronic stimulation by promoting RIP2 degradation.

## Results

### RNAi screen identifies novel regulators of NOD2 signalling

**Figure 1:**
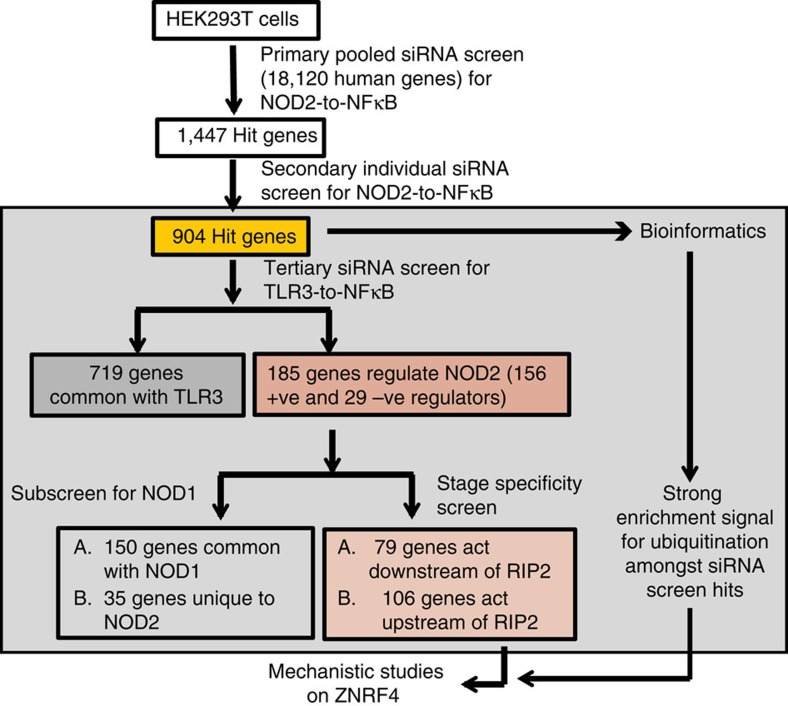
Genome-wide RNAi screening and results analysis. RNAi screen methodology to identify novel regulatory genes regulating NOD2-induced NF-κB activation.

To identify the human genes negatively regulating NOD2-induced NF-κB activation (NOD2-to-NF-κB), we performed an *in vitro* human genome-wide RNAi screen in HEK293T cells stably expressing both human NOD2 and an NF-κB target promoter-driven GFP reporter (293T-NOD2-NF-κB-GFP) and stimulated with the ligand, MDP (Fig. 1). Assay details, RNAi screen methodology and on-target specificity validation approaches are given in the Methods section and Supplementary Fig. 1a–d. We performed a multitiered RNAi screen (Fig. 1) involving an initial whole-genome screen for regulators of NOD2-to-NF-κB, and a subsequent filtering screen with the obtained hits on the unrelated Toll-like receptor 3 (TLR3) (unrelated endosomal/intracellular PRR)-mediated NF-κB activation, to exclude generic NF-κB regulators. The screen recovered several known regulators of NOD2-to-NF-κB signalling including RIP2, IKKα, NEMO, RELA, ERBIN and CYLD, highlighting the reliability of our approach (Supplementary Data 1). Analysis of the screen hits also revealed that several of the newly identified screen hits interacted with many previously known components of NOD2 signalling network (Supplementary Fig. 1e). We identified 185 genes (156 positive and 29 negative regulators) that are considered specific regulators of NOD2 signalling (Supplementary Data 1), which did not impact on the TLR3 pathway. Using a secondary assay involving RIP2 ectopic expression, we determined that 79 and 106 hit genes (out of 185 total hits) likely act, respectively, downstream and upstream of RIP2 (Fig. 1 and Supplementary Data 1). Among the 185 NOD2 RNAi screen hits, 150 (81%) were also involved in NOD1-mediated NF-κB activation (Fig. 1 and Supplementary Data 1), implying that the remaining 35 genes play potential MDP-NOD2 module-specific regulatory roles.

### ZNRF4 is a negative regulator of NOD2 and NF-κB signalling

**Figure 2:**
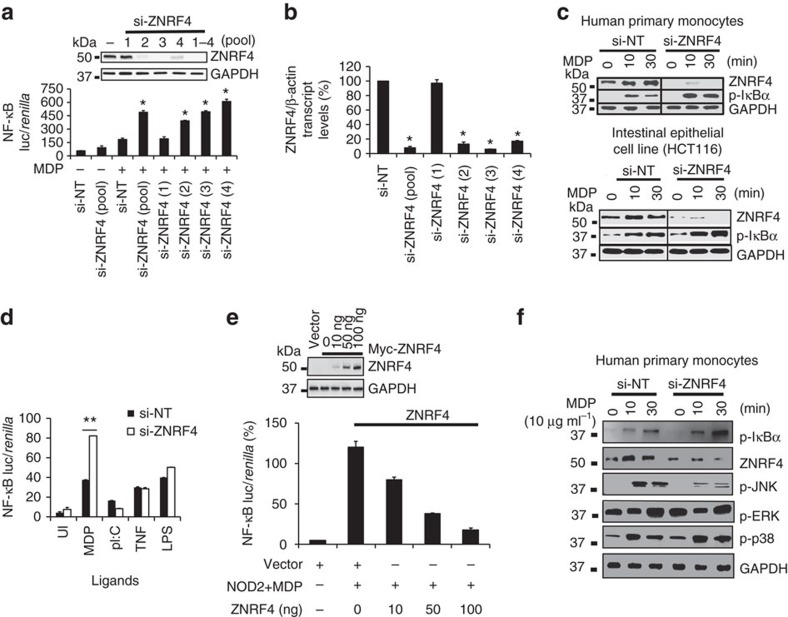
ZNRF4 negatively regulates NOD2-mediated NF-κB activation. (**a**) NF-κB luciferase activity in unstimulated or MDP-stimulated HEK293T-NOD2 cells transfected with si-NT or siRNA targeting *ZNRF4* (four unique siRNAs (1, 2, 3 or 4) and pooled siRNA). Immunoblotting (inset panel) confirmed gene silencing. *P≤0.05 compared with MDP-stimulated si-NT values. (**b**) qRT–PCR analysis of *ZNRF4* mRNA levels following *ZNRF4* gene silencing in HEK293T cells (normalized with β-actin). (**c**) Immunoblot to show the effects of ZNRF4 gene silencing on MDP-induced NF-κB activation (by measuring p-IκBα levels) in human primary monocytes (CD14+, top panel) and HCT116 cell line (bottom panel). (**d**) NF-κB luciferase activity in HCT116 cells transfected with si-NT or si-ZNRF4, and stimulated with the ligands MDP (10 μg ml^−1^), pIC (5 μg ml^−1^), TNF (10 ng ml^−1^) or lipopolysaccharide (100 ng ml^−1^) for 24 h. (**e**) NF-κB luciferase activity in MDP-treated HEK293T-NOD2 cells, transfected with vector control or indicated amounts of plasmid encoding *ZNRF4*. Inset panel: expression levels of ZNRF4. (**f**) Immunoblot to show the effect of ZNRF4 gene silencing on MDP-induced IκBα, extracellular signal-regulated kinase (ERK1/2), p38 and Jun N-terminal kinase phosphorylation in human primary monocytes (CD14+). **P≤0.01, *P≤0.05. Significance was assessed using Student’s *t*-test. Data are representative of (**c** and **f**) three independent experiments with similar results or represent the mean±s.d. of (**a**,**b**,**d**,**e**) three independent experiments. si, siRNA; si-NT, non-targeting negative control siRNA; UI, uninduced.

Enrichment analysis of the screen hits revealed a significant enrichment of ubiquitination-related genes (Supplementary Fig. 2a). Among these, 21 genes were identified as novel negative regulators of NOD2 signalling that included ubiquitin ligases, their adaptors or their interacting partners (Supplementary Fig. 2b). Consistent with our goal of identifying novel ubiquitination-mediated negative regulation of NOD2 signalling, we initially focused on investigating the role of newly identified E3-ubiquitin ligases. Among the six newly identified E3-ubiquitin ligase genes, silencing of the E3-ubiquitin ligase *ZNRF4* (also termed nixin)^28^ showed an increase of the NF-κB response to MDP stimulation (Supplementary Data 1). ZNRF4 was previously reported as an ER-anchored RING-finger containing E3-ubiquitin ligase (490 amino acids), with its catalytic ligase domain facing the cytoplasm^28^. The only known function of ZNRF4 is that it can induce degradation of the chaperone calnexin, which led us to reason that ZNRF4 might be regulating NOD2 pathway through its ability to mediate degradation of signalling proteins. Because the functional involvement of ZNRF4 in the regulation of immune signalling is currently unknown, we chose to further characterize its function in NOD2 signalling. Using an NF-κB luciferase reporter-based assay in HEK293T cells ectopically expressing the *NOD2* gene, we validated that *ZNRF4* silencing significantly increased MDP-induced NF-κB activation (2.6-fold using the pooled short interfering RNA (siRNA), *P*<0.01; Fig. 2a). ZNRF4 knockdown was confirmed at the protein (Fig. 2a) and mRNA levels (Fig. 2b), using three independent siRNAs targeting unique regions of ZNRF4 and their pool. We further determined that ZNRF4 negatively regulated MDP-induced NF-κB activation in human primary monocytes (CD14+) (Fig. 2c, upper panel), and also in the intestinal epithelial cell line HCT116 (Fig. 2c, lower panel), two human cell types that endogenously express *NOD2*. Consistent with this, DNA-binding activity of NF-κB p65 was markedly increased in *ZNRF4*-silenced HCT116 cells on MDP stimulation (Supplementary Fig. 2c). Importantly, *ZNRF4* silencing did not increase TLR3-, TLR4- or tumour necrosis factor (TNF)-induced NF-κB activation (Fig. 2d), such that ZNRF4 demonstrates specificity in its negative regulation. Neither silencing nor overexpression of *ZNRF4* altered cell viability.

**Figure 3:**
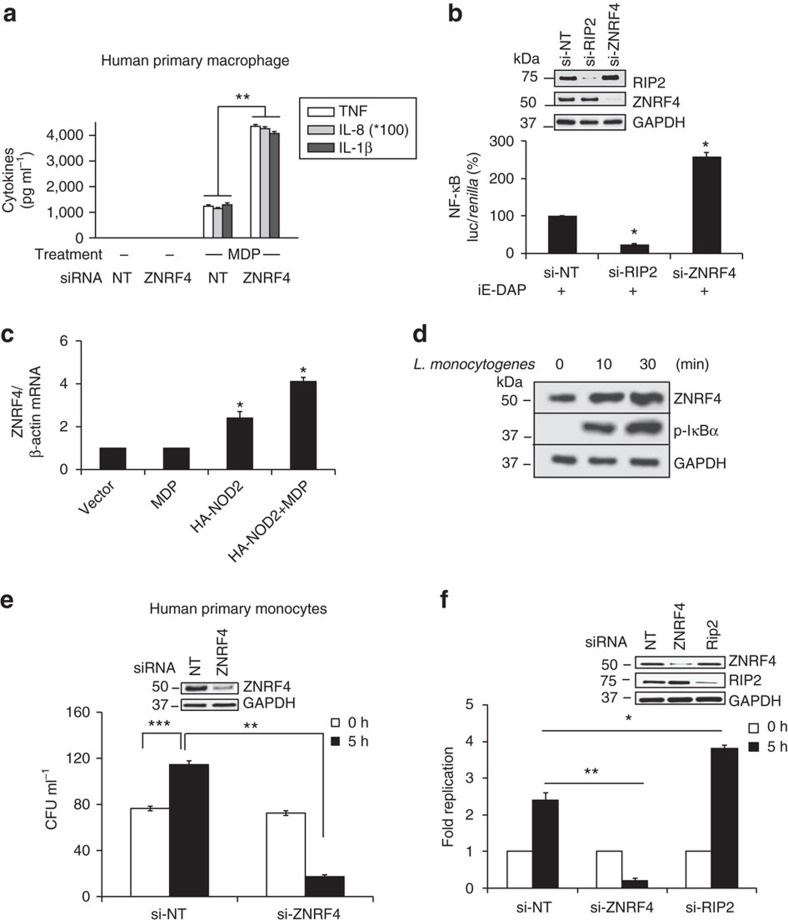
ZNRF4 negatively regulates MDP-induced proinflammatory response and host control of bacterial infection. (**a**) IL-8, TNF and IL-1β secretion in ZNRF4 knockdown human primary macrophages following MDP treatment. (**b**) NF-κB luciferase activity in iE-DAP-stimulated HEK293T-NOD1 cells transfected with si-NT, si-ZNRF4 or si-RIP2. (**c**) Quantitative RT–PCR analysis to measure transcript levels of ZNRF4 in MDP-treated HEK293T cells with and without NOD2 overexpression. Data are normalized to β-actin gene. (**d**) Immunoblot to measure the protein expression levels of ZNRF4 in human primary monocytes with *L. monocytogenes* stimulation. (**e**,**f**) CFU assay to measure the intracellular bacterial load in (**e**) human primary monocytes and (**f**) HCT116 cells transfected with si-NT, or si-ZNRF4 on bacterial infection. Si-RIP2=positive control. The results of (**e**,**f**) are represented as CFU ml^−1^ or as the fold replication of bacteria (at 5 h after infection in comparison with 0 h) respectively. Knockdown efficiency is shown by western blot (inset). *P<0.05 and **P<0.01. Significance was assessed using (**a**–**c**) Student’s *t*-test or (**e**,**f**) one-way analysis of variance (ANOVA). Data represent mean±s.e.m. from (**a**) three independent experiments (compared with si-NT sample with MDP stimulation) or mean±s.d. from (**b**,**c**,**e**,**f**) three independent experiments or representative of (**d**) three independent experiments with similar results. si, siRNA; si-NT, non-targeting negative control siRNA.

Complementing gene silencing-based results, overexpression of *ZNRF4* reduced NOD2-induced NF-κB activation in a dose-dependent manner (Fig. 2e). *ZNRF4* silencing also decreased NOD2-induced Jun N-terminal kinase (JNK) activation, but did not affect p38 and extracellular signal-regulated kinase 1/2 activation in human primary monocytes (Fig. 2f). We next examined whether ZNRF4 regulates NOD2-dependent proinflammatory cytokine or chemokine secretion. Silencing of ZNRF4 led to increased secretion of interleukin-8 (IL-8), TNF and IL-1β in MDP-treated primary human monocyte-derived macrophages (Fig. 3a). We further found that *ZNRF4* silencing also increased NOD1-mediated NF-κB activation (Fig. 3b) and cytokine secretion (Supplementary Fig. 3a).

We next investigated whether ZNRF4 expression is sensitive to the activation of the NOD2 pathway. Treatment of HEK293T cells (which do not express detectable levels of endogenous NOD2) with empty vector or MDP alone did not cause any upregulation of ZNRF4 transcripts. However, overexpression of NOD2 alone caused transcriptional upregulation of ZNRF4 transcripts, which was further enhanced with MDP stimulation (Fig. 3c). We also observed that MDP stimulation (Fig. 2c, top panel) and *Listeria monocytogenes* infection (Fig. 3d) upregulated ZNRF4 at the protein level in human primary monocytes. These observations demonstrate that ZNRF4 expression is upregulated on NOD2 stimulation, potentially to negatively regulate NOD2-induced signalling and cytokine secretion.

### ZNRF4 negatively regulates host anti-bacterial defense

We subsequently investigated the biological importance of ZNRF4 in the anti-bacterial response of host cells. Notably, ZNRF4 depleted human primary monocytes displayed a significantly enhanced ability to control *L. monocytogenes* replication (Fig. 3e). As intestinal epithelial cells are the first line of defense on oral *L. monocytogenes* exposure, we subsequently examined the ZNRF4 regulation of *L. monocytogenes*-induced NF-κB activation and bacterial replication in HCT116 cells. *L. monocytogenes* infection induced greater NF-κB activation (4.6-fold, *P*<0.05; Supplementary Fig. 3b) and cytokine responses (Supplementary Fig. 3c) in ZNRF4-silenced HCT116 cells, compared with control cells. Similar to primary monocytes, ZNRF4-depleted intestinal cells also displayed a significantly enhanced ability to control *L. monocytogenes* replication (Fig. 3f). These findings demonstrate that ZNRF4 expression attenuates NOD1/NOD2-mediated innate immune responses to *L. monocytogenes* infection.

### ZNRF4 inhibits NOD2 signalling via RIP2 degradation

**Figure 4:**
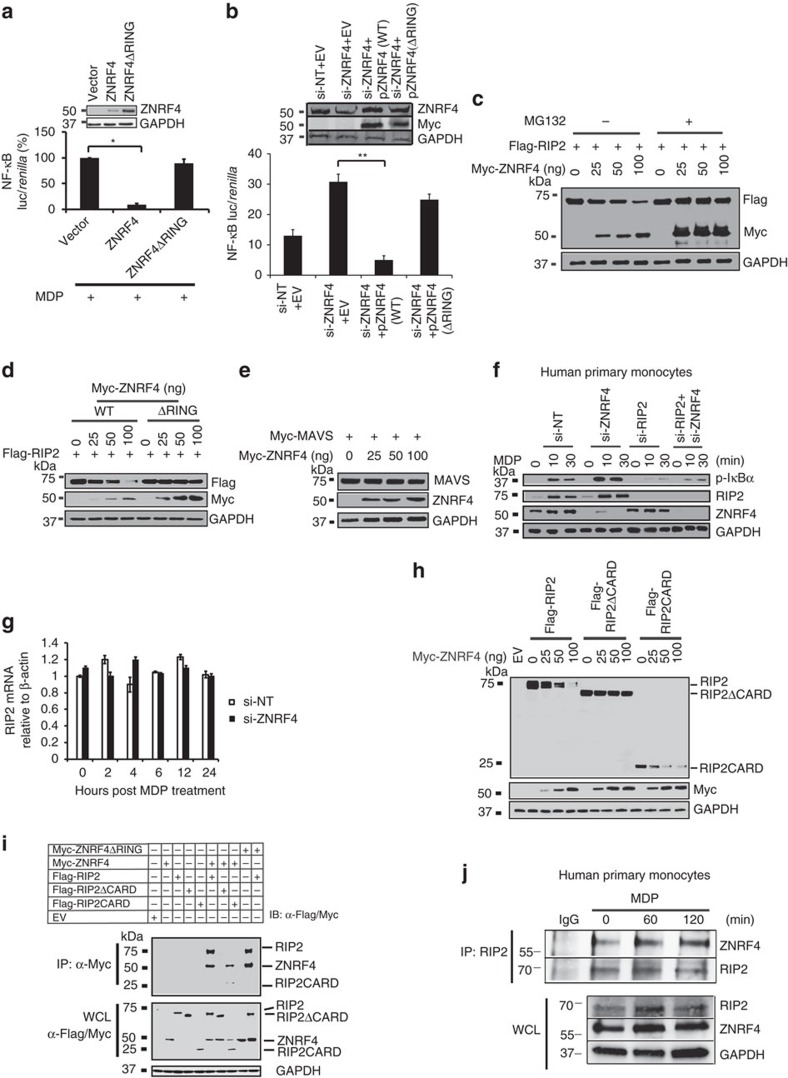
ZNRF4 interacts with and promotes degradation of RIP2. (**a**) NF-κB luciferase reporter activity (relative to internal control *Renilla* luciferase activity) in HEK293T-NOD2 cells transfected with WT ZNRF4 or RING mutant (ZNRF4ΔRING) plasmids, following MDP stimulation. Immunoblotting confirmed the expression of various proteins (inset panel). (**b**) NF-κB luciferase activity in MDP-stimulated HCT116 cells transfected with si-NT or si-ZNRF4 (targeting the 3′-UTR of ZNRF4), followed by transfection with plasmid (Myc-tagged) encoding either WT *ZNRF4* (pZNRF4(WT)) or RING mutant of ZNRF4 (pZNRF4(ΔRING)). All NF-κB luciferase values were normalized with *Renilla* luciferase activity. (**c**–**e**) Immunoblot of extracts of HEK293 cells transfected with expression plasmids for (**c**,**d**) Flag-RIP2 (250 ng) and increasing amounts of (**c**) Myc-ZNRF4 (25–100 ng) and treated with DMSO or MG-132 or (**d**) either WT or RING mutant (ΔRING) Myc-ZNRF4 and (**e**) HA-MAVS and Myc-ZNRF4. (**f**) Human primary monocytes (CD14+) treated with *ZNRF4*-specific siRNA (si-ZNRF4), si-RIP2, si-ZNRF4+siRIP2 or si-NT and stimulated with MDP (10 μg ml^−1^) for the indicated times and immunoblotted for the indicated proteins. GAPDH=loading control. (**g**) Quantitative RT–PCR analysis of *RIP2* mRNA in si-ZNRF4 or si-NT treated HCT116 cells following MDP stimulation. (**h**) Immunoblot of extracts of HEK293 cells expressing Flag-RIP2, Flag-RIP2ΔCARD or Flag-RIP2CARD (250 ng each) with Myc-ZNRF4. (**i**) Co-immunoprecipitation (top panel) and lysates (bottom panel) of HEK293 cells expressing Flag-RIP2, Flag-RIP2ΔCARD or Flag-RIP2CARD along with Myc-ZNRF4 or Myc-ZNRF4ΔRING. (**j**) Co-immunoprecipitation with RIP2 (top panel) and lysates (bottom panel) of human primary monocytes (CD14+) cells treated with MDP for various time points. (**a**,**b**,**g**) Data represent mean±s.d. of triplicate samples and are representative of three independent experiments. *P<0.05 and **P<0.01. Significance was assessed using Student’s *t*-test. Experiments (**c**–**f** and **h**–**j**) were repeated three times with similar results. Representative blots are shown. EV, vector control; si, siRNA; si-NT, non-targeting negative control siRNA.

We next sought to determine the mechanism by which ZNRF4 regulates NOD2 signalling. Because ZNRF4 is an E3-ubiquitin ligase, we hypothesized that it may regulate NOD2 responses by ubiquitinating a signalling pathway component protein. We first investigated whether the RING domain of ZNRF4 is required for its effect on NOD2 signalling, by using the catalytically inactive double point mutant (H329W, H332W; denoted as ZNRF4ΔRING)^28^. While overexpression of ZNRF4 wild-type (WT) construct reduced NOD2-mediated NF-κB activation, the ring domain mutant did not, demonstrating that the ligase activity of ZNRF4 is crucial to its regulatory function on NOD2 signalling (Fig. 4a). Rescue experiments also showed that overexpression of WT but not the RING mutant ZNRF4 led to a reduction in the enhanced NOD2-induced NF-κB activation observed in ZNRF4 knockdown (using siRNA targeting the 3′-untranslated region (UTR) of ZNRF4) cells (Fig. 4b). Because ZNRF4 is a negative regulator, and regulates both NOD1 and NOD2 signalling, we further speculated that it might function by degrading a signalling component protein common to both NOD1 and NOD2 pathways such as RIP2. Consistent with this, the stage-specific assays indicated that ZNRF4 regulates NOD2 signalling at the level or downstream of RIP2 (Supplementary Data 1) in this assay. Moreover, MDP treatment is previously reported to induce degradation of RIP2 (ref. 19), which was also reproduced in our experiments using human primary monocytes (Supplementary Fig. 3d). Cycloheximide (CHX) chase assay further substantiated this result (Supplementary Fig. 3e). As RIP2 is the major known signal integrator for both NOD1 and NOD2 pathways, and NOD2 activation downregulates RIP2 expression, we hypothesized that ZNRF4 might target RIP2 for regulating NOD2 pathway. Thus, we next investigated the effect of ZNRF4 expression on the steady-state levels of RIP2. As seen in Fig. 4c, ectopic expression of ZNRF4 significantly reduced the protein levels RIP2 in a dose-dependent manner. Pretreatment of cells with the proteasomal inhibitor MG132 rescued RIP2 degradation, confirming that the observed RIP2 degradation by ZNRF4 was proteasome-dependent. The inactive RING domain mutant of ZNRF4 did not degrade RIP2, indicating that the ligase activity is needed for ZNRF4 to degrade RIP2 (Fig. 4d). In contrast, ZNRF4 did not regulate the levels of MAVS, the signalling adaptor of unrelated cytosolic anti-viral PRR, RIG-I (Fig. 4e). Conversely, knockdown of endogenous ZNRF4 in human primary monocytes increased the cellular protein level of endogenous RIP2 in comparison with negative control siRNA-treated cells (Fig. 4f). Accordingly, this correlated with enhanced activation of the NF-κB pathway (shown by increased levels of phospho (p)-IκBα). As expected, the increase in MDP-induced NF-κB activation seen in ZNRF4-silenced cells was ablated in ZNRF4 and RIP2-double-knockdown cells (Fig. 4f). Consistent with our demonstration that ZNRF4-mediated regulation of RIP2 is occurring at the protein level through a proteasome-dependent mechanism (Fig. 4c), silencing of ZNRF4 did not alter the transcription of RIP2 (Fig. 4g). The CARD domain-deleted RIP2 (RIP2ΔCARD) was not degraded by ZNRF4, indicating that the CARD domain of RIP2 (ref. 29) was required for ZNRF4-mediated degradation (Fig. 4h). Consistently, a truncation mutant of RIP2 expressing the CARD domain alone (RIP2CARD) was degraded by ZNRF4 (Fig. 4h). Collectively, ZNRF4 degrades RIP2 protein by targeting the RIP2CARD domain in an E3-ubiquitin ligase activity-dependent manner.

### ZNRF4 interacts with RIP2

Because ZNRF4 was found to induce degradation of RIP2, we questioned whether it mediates this function by interacting with RIP2. Co-immunoprecipitation experiments in HEK293 cells ectopically expressing ZNRF4 and RIP2 identified that both the WT and RING mutant of ZNRF4 interacted with RIP2 (Fig. 4i). Domain mapping identified that the CARD domain of RIP2 was required for its interaction with ZNRF4. While RIP2ΔCARD did not interact with ZNRF4, RIP2CARD alone interacted (Fig. 4i).

We subsequently confirmed that endogenous ZNRF4 and RIP2 also interacted with each other, and determined the kinetics of their interaction by a time-course co-immunoprecipitation assay with and without MDP treatment in human primary monocytes (Fig. 4j). There was a low level of ZNRF4 and RIP2 interaction in unstimulated human monocytes (Fig. 4j). However, an increase in the association between the two proteins was detected following NOD2 stimulation in human monocytes (Fig. 4j). These data establish that interaction of ZNRF4 with RIP2 increases in primary monocytes on NOD2 stimulation, and that ZNRF4–RIP2 interactions are mediated through the CARD domain of RIP2.

### ZNRF4 promotes K48-linked ubiquitination of RIP2

**Figure 5:**
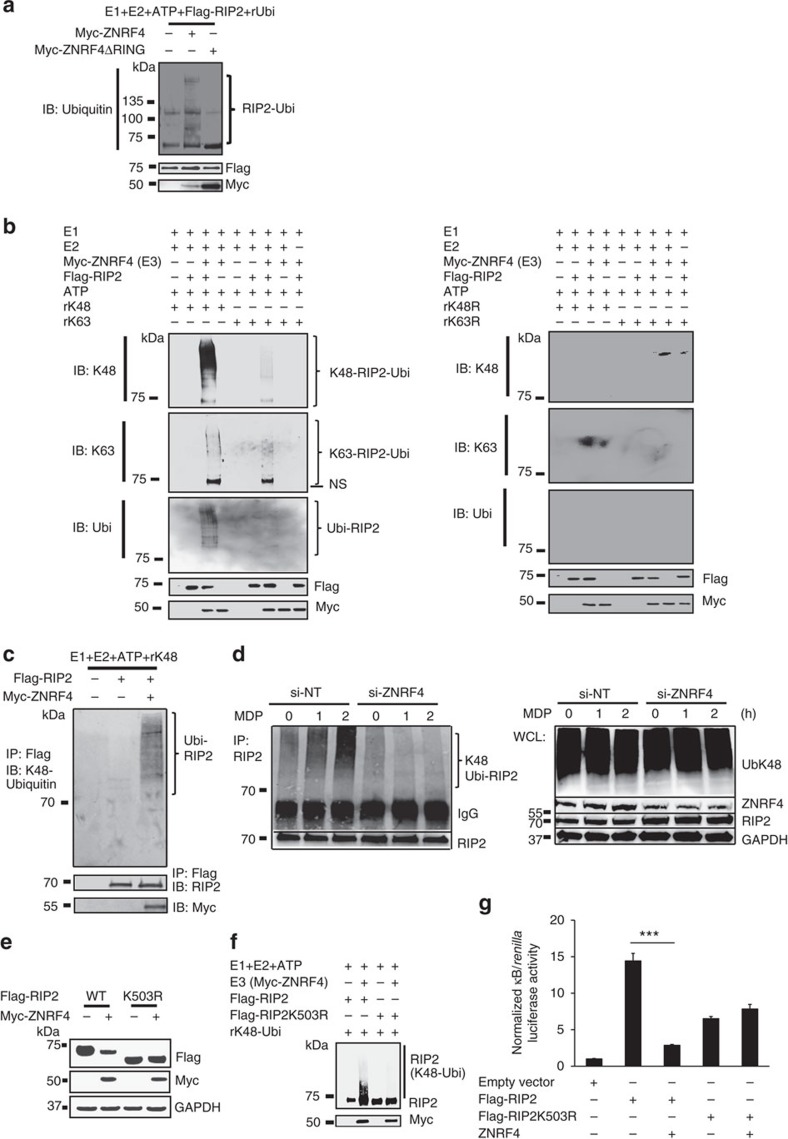
ZNRF4 mediates RIP2 ubiquitination via K48-specific ubiquitin linkages. Immunoblot analysis of (**a**) total ubiquitination and (**b**) K48-mediated and K63-mediated ubiquitination of Flag-RIP2 assessed by *in vitro* ubiquitination assay with various combinations of a mixture of E1 enzyme, E2 enzymes, ATP, recombinant Ub (rUbi), recombinant ubiquitin K48/K48R, K63/K63R, Myc-ZNRF4 (100 ng) or Myc-ZNRF4ΔRING (100 ng). NS represents nonspecific band. (**c**) K48-mediated ubiquitination of RIP2 by ZNRF4 assessed following immunoprecipitation of RIP2 from the *in vitro* ubiquitination reaction mix containing a mixture of E1 and E2 enzymes, ATP, recombinant ubiquitin K48 and Myc-ZNRF4. (**d**) HCT116 cells were transfected with either NT-siRNA or ZNRF4-siRNA. After 2 days, cells were transfected with HA-ubiquitin. The next day, cells were stimulated with MDP for various time points and cell lysates were subjected to immunoprecipitation using and anti-RIP2 antibody and immunoblotted with anti-K48-ubiquitin antibody. (**e**) Immunoblot analysis of extracts of HEK293 cells transfected with expression plasmids of Flag-RIP2 or Flag-RIP2K503R mutant in the presence or absence of Myc-ZNRF4. (**f**) Immunoblot analysis of K48-mediated, ubiquitination of Flag-RIP2 or Flag-RIP2K503R mutant assessed by *in vitro* ubiquitination assay with various combinations of a mixture of E1 enzyme, E2 enzymes, ATP, recombinant ubiquitin K48 and Myc-ZNRF4. (**g**) Activation of NF-κB promoter in HCT116 cells transfected with NF-κB luciferase reporter and expression vectors of Flag-RIP2 or Flag-RIP2K503R mutant and ZNRF4 (untagged). The results are normalized to that of *Renilla* luciferase. (**g**) Data represent the mean±s.d. of three independent experiments performed in triplicate. ***P<0.001. Significance was assessed using Student’s *t*-test. Experiments (**a**–**f**) were repeated three times with similar results. Representative blots are shown.

Since ZNRF4 regulates RIP2 levels through its RING domain catalytic activity, we examined whether ZNRF4 regulates NOD2 signalling through direct ubiquitination of RIP2, first using cell-free *in vitro* ubiquitination assays. A mixture containing recombinant Flag-RIP2, Myc-ZNRF4, Myc-ZNRF4ΔRING and different E1 and E2 enzymes was incubated separately with WT Ub, UbK48, UbK63, UbK48R and UbK63R. As seen in Fig. 5a, WT ZNRF4 induced ubiquitination of RIP2 when incubated with WT Ub; however, the RING mutant of ZNRF4 (Myc-ZNRF4ΔRING) was unable to induce ubiquitination of RIP2. Further experiments revealed that ZNRF4 induced K48-, not K63-, dependent ubiquitination on RIP2 (Fig. 5b). Accordingly, ZNRF4-induced ubiquitination on RIP2 was lost when the K48 mutant (K48R) ubiquitin was used (Fig. 5b). We further confirmed the specificity of RIP2 as ubiquitination substrate for ZNRF4 by immunoprecipitating RIP2 following denaturation of the *in vitro* ubiquitination reaction complex (Fig. 5c). Additionally, we also found that K48 ubiquitination of endogenous RIP2 was significantly diminished in ZNRF4-knockdown cells following MDP stimulation (Fig. 5d). These results demonstrate that ZNRF4 promotes K48-linked ubiquitination of RIP2.

As E3 ligases attach ubiquitin chains to the lysine residues of target proteins, we aimed to identify the lysines on RIP2 that are potentially involved in its regulation by ZNRF4. Since the CARD domain of RIP2 alone was sufficient for degradation, we reasoned that the lysine(s) targeted by ZNRF4 lies in the CARD domain. We individually mutated the lysine residues within the CARD domain of RIP2 to arginine, and assessed whether the mutant RIP2 are degraded/ubiquitinated by ZNRF4. Mass spectrometric analysis also identified lysine 503 as an ubiquitination site of RIP2 (Supplementary Data 2). Whereas ZNRF4 mediated the degradation of WT RIP2, RIP2 mutated at lysine-503 (RIP2-K503R) was not efficiently degraded by ZNRF4 (Fig. 5e). Consistent with this, *in vitro* ubiquitination assay revealed that ZNRF4 was unable to ubiquitinate the RIP2-503R mutant (Fig. 5f). Additionally, there was no significant difference in the activation status of NF-κB when RIP2-K503R was coexpressed with ZNRF4 (Fig. 5g). RIP2-K503R mutant retained its ability to interact with major signalling components of NOD2 signalling pathway such as TAK1 and NEMO, as well as ZNRF4, similar to WT RIP2 (Supplementary Fig. 3f,g, respectively). Thus, ZNRF4-induced K48-linked ubiquitination of RIP2 depends on a lysine at position 503 in the CARD domain of RIP2.

### RIP2 colocalizes with ZNRF4 at the ER

**Figure 6:**
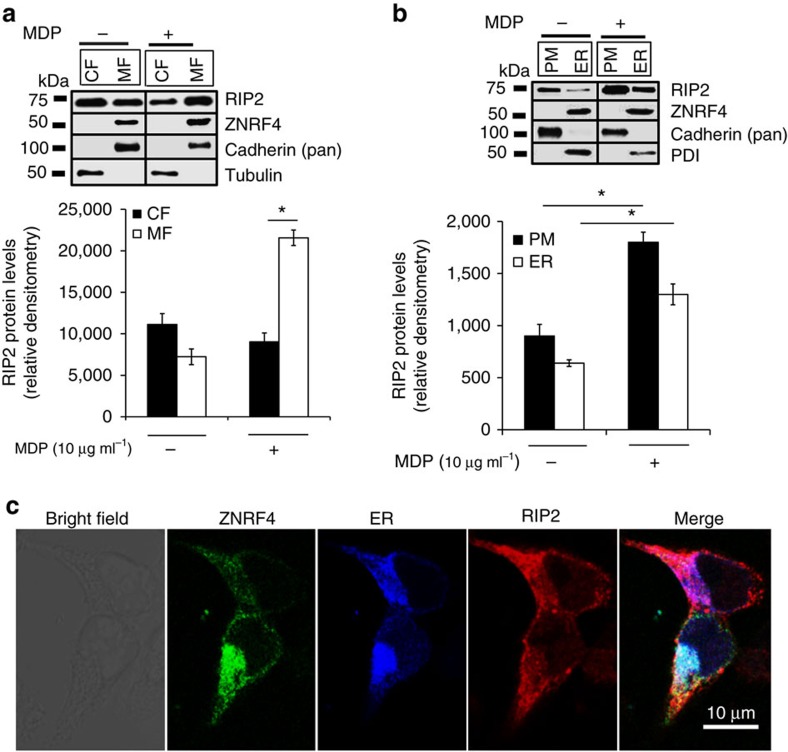
RIP2 colocalizes with ZNRF4 at the ER. (**a**,**b**) Immunoblot analysis for RIP2 from various cellular fractions of HCT116 cells stimulated with MDP for 30 min. The cells were fractionated to separate (**a**) cytoplasmic (CF), membrane (MF) or (**b**) plasma membrane (PM), endoplasmic reticulum (ER) fractions, respectively. Equal amount of each fraction (30 μg) was analysed by western blot. Relative densitometric data (using ImageJ) of RIP2 levels in various fractions is shown in the bottom panels (*n*=3). (**c**) Confocal microscopy of HCT116 cells expressing RIP2-HA (red), YFP-ZNRF4 (green) and pmTurquoise2-ER (ER marker-blue). EV, vector control. Images were acquired at × 63 optical magnification. Scale bars, 10 μm. Western blots and confocal images are representative of one of three independent experiments that were conducted. *P<0.05. Significance was assessed using Student’s *t*-test. si-NT, non-targeting negative control siRNA. Protein disulfide isomerase (PDI)=marker for ER; cadherin (pan)=marker for PM.

To gain additional insight into ZNRF4–RIP2 interactions within the cell, we next sought to determine the potential subcellular locations where these proteins functionally interact. While ZNRF4 is reported to reside in the ER^28^, previous studies have shown that RIP2 localizes to either the cytoplasm (in unstimulated cells) or plasma membrane (PM) (on MDP stimulation)^30^. We first reinvestigated the subcellular localization of ZNRF4 and RIP2 using a sensitive cell fractionation approach in HCT116 cells with or without MDP stimulation. Figure 6a shows that RIP2 is present in both cytoplasmic fractions and membranous fractions (mixture of PM and ER) in unstimulated WT cells. Further separation of the membrane fraction into ER and PM revealed a previously undefined localization pattern for RIP2: a fraction of RIP2 was also localized to the ER (Fig. 6b). On MDP treatment, increased RIP2 translocation to the PM was observed, although a fraction of RIP2 was still present in the ER. As reported previously, ZNRF4 could be detected only in the ER fraction both in unstimulated and MDP-stimulated conditions (Fig. 6b). The purity of fractions was assessed by detecting organelle-specific markers for ER (PDI) and PM (pan-cadherin). We further confirmed the localization of ectopically expressed RIP2 and ZNRF4 at the ER using a confocal microscopy-based independent approach (Fig. 6c and Supplementary Fig. 4a), Mander’s overlap coefficients (*N*≥50) of RIP2/ER is 0.76±0.05, ZNRF4/ER is 0.75±0.04 and that of RIP2/ZNRF4 is 0.65±0.12). Notably, the CARD-deleted RIP2 was absent from the membrane fraction (Supplementary Fig. 4b). Collectively, these data demonstrate that a notable fraction of RIP2 localizes to the ER, suggesting the ER as the most likely site for the ZNRF4–RIP2 interactions that are critical for proper NOD2 regulation.

### RIP2 degradation by ZNRF4 promotes NOD2-induced tolerance

**Figure 7:**
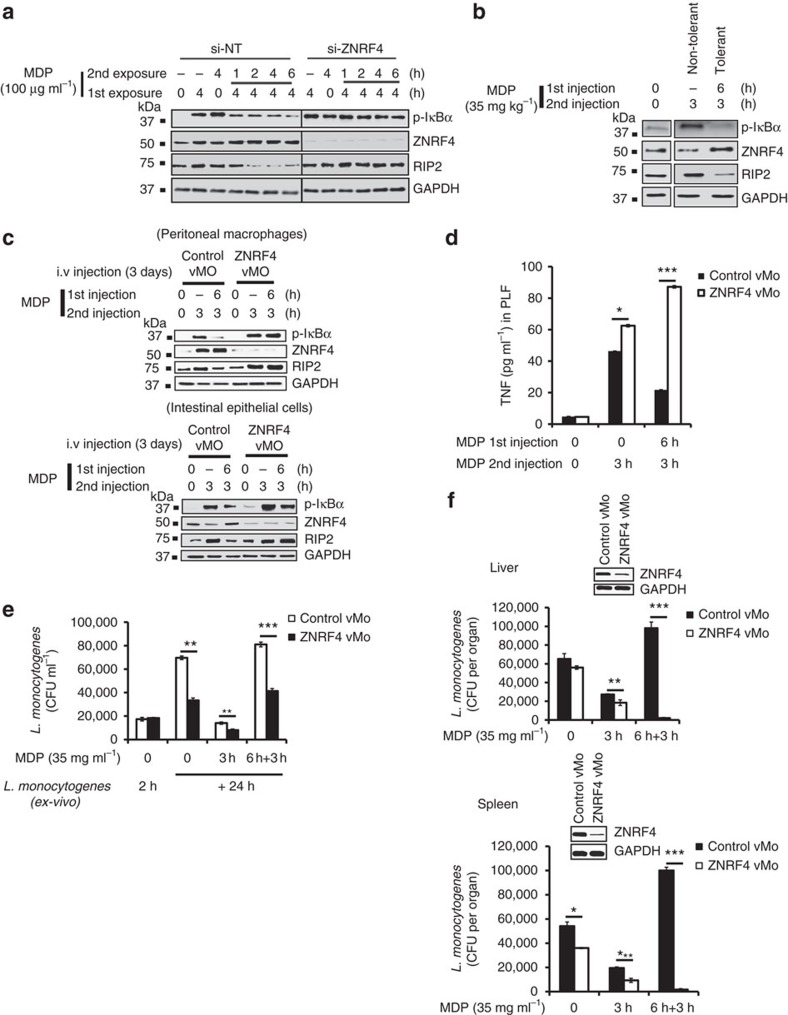
ZNRF4-mediated RIP2 degradation is needed for the NOD2-induced tolerance. (**a**–**c**) Immunoblot analysis of (**a**) differentiated THP-1 cells that were exposed to *ZNRF4*-siRNA (si-ZNRF4) or non-targeting siRNA (si-NT) followed by MDP pre-treatment, and re-treatment with MDP for the indicated time intervals to induce NOD2 tolerance. (**b**) Murine peritoneal macrophages during MDP tolerance induction *in vivo*. Thioglycollate-treated mice (C57BL/6) were given MDP (35 mg kg^−1^) intraperitoneally: MDP-tolerant group first received an MDP injection for 6 h and then a second injection for 3 h. The non-tolerant group received only the second injection of MDP. Control mice received thioglycollate only. After the second MDP stimulation, peritoneal cavity macrophages were collected by lavage and lysed for western blot analysis. (**c**) *In vivo Znrf4* knockdown abrogates NOD2 tolerance induction. Thioglycollate-treated mice received intravenously either vMO targeting murine ZNRF4 (ZNRF4 vMO) or vMO standard control (control vMO), followed by the MDP treatment on day 4 for the indicated time points, and tissues (murine peritoneal macrophages and intestinal epithelial cells) were collected. (**c**) Western blots on lysates or (**d**) ELISA for TNF in peritoneal lavage fluid was performed. (**e**,**f**) Thioglycollate-treated mice (*n*=3 mice per group) received intravenously ZNRF4 vMO or control vMO. On the fourth day, MDP- or endotoxin-free PBS was injected intraperitoneally for the indicated time points. After the last injection of MDP, (**e**) peritoneal lavage was collected, and infected *ex vivo* with *L. monocytogenes* (MOI*=*1) for the indicated time points. The cells were lysed, and the bacterial load was measured as CFU ml^−1^ or (**f**) mice were infected intraperitoneallywith *L. monocytogenes*. At 24 h after infection, mice were killed, and the liver/spleen were analysed for bacteria quantification. The bacterial load was measured as CFU per organ. Western blot (inset) shows the knockdown efficiency. *P<0.05, **P<0.01 and ***P<0.001. Significance was assessed using Student’s *t*-test. Data are representative of (**a**,**b**,**e**) three independent experiments or (**c**,**d**,**f**) two independent experiments with similar results. (**c**,**f**) Mean±s.d. of (**c**) four or (**f**) three mice. si, siRNA; si-NT, non-targeting negative control siRNA.

Prolonged activation of NOD2 has been demonstrated to induce tolerance to MDP; however, the mechanisms underlying MDP-induced self-tolerance have yet to be fully defined. Particularly, although RIP2 degradation has been reported during NOD2-induced tolerance^19^, the regulatory mechanism governing this process is not known. Since our experiments identified ZNRF4 as a key negative regulator of NOD2 signalling, we questioned whether ZNRF4-mediated RIP2 degradation is also involved in establishing tolerance in innate immune cells after chronic NOD2 stimulation. For this, we initially utilized human monocytic THP-1 cells as the model system. After acute MDP exposure for 4 h, re-treatment of THP-1 cells with MDP resulted in reduced p-IκBα levels in comparison with that observed with acute MDP treatment (Fig. 7a). This observation was consistent with a previous report^19^. In contrast, ZNRF4-silenced cells showed a sustained activation of NF-κB compared with control cells under these same conditions, demonstrating that ZNRF4 expression was needed for NOD2-induced tolerance. Consistent with the impaired downregulation of NF-κB signalling, we found that ZNRF4-deficient cells failed to attenuate RIP2 protein expression observed under NOD2-induced tolerance conditions (Fig. 7a). These results were further validated by restimulating MDP-pretreated cells with *L. monocytogenes* (Supplementary Fig. 5a). Moreover, ZNRF4-mediated RIP2 degradation was also essential for MDP tolerance induction in human primary CD14+ monocytes (Supplementary Fig. 5b). Therefore, we identify that the RIP2 degradation which contributes to the dramatically diminished signalling observed after chronic NOD2 stimulation is dependent on ZNRF4.

### ZNRF4 is required for NOD2-induced tolerance *in vivo*

We next investigated if ZNRF4 and RIP2 expression levels are modulated during NOD2 tolerance induction *in vivo*, utilizing a previously reported^19^ mouse model system of NOD2 tolerance. Mice were injected intraperitoneally with MDP (35 mg kg^−1^) and were subsequently challenged with a second MDP injection after 6 h and peritoneal macrophages were then collected. Consistent with the results obtained using cell line and primary cells, induction of NOD2 tolerance *in vivo* was accompanied by enhanced expression of ZNRF4, and lower levels of RIP2, p-IκBα (Fig. 7b) in peritoneal macrophages in comparison with control PBS-treated mice.

To establish convincingly the specific requirement of ZNRF4 in NOD2 tolerance induction *in vivo*, we utilized *vivo*-morpholino (vMO)-mediated^31^ systemic *Znrf4* knockdown in mice. *Znrf4* was transiently knocked down by intravenous administration of vMO directed against *Znrf4*. Mice were then injected intraperitoneally with MDP and were subsequently challenged with a second MDP injection after 6 h. vMO treatment resulted in efficient knockdown of *Znrf4* in peritoneal macrophages and intestinal epithelial cells (Fig. 7c). Consistent with the *in vitro* results, we found that *Znrf4* knockdown mice lost the ability to induce NOD2 tolerance; they showed higher levels of RIP2 and p-IκBα in peritoneal macrophages and intestinal epithelial cells (Fig. 7c). Accordingly, the proinflammatory cytokines TNF and IL-6 were higher in peritoneal fluid from *Znrf4* knockdown animals under acute NOD2 stimulation and failed to decrease with chronic NOD2 stimulation (Fig. 7d and Supplementary Fig. 5c, respectively).

We next examined the physiological relevance of ZNRF4-mediated RIP2 regulation during NOD2-tolerant conditions in the context of bacterial infection. Following *Znrf4* knockdown and NOD2 tolerance induction, mouse peritoneal macrophages were infected *ex vivo* with *L. monocytogenes* and the intracellular bacterial load was assessed. NOD2-tolerized control-morpholino-treated cells showed impaired ability to control *L. monocytogenes* infection. However, NOD2-tolerized *Znrf4* knockdown macrophages showed significantly enhanced ability to control bacterial infection (Fig. 7e). Accordingly, NOD2-tolerized *Znrf4* knockdown peritoneal macrophages showed increased expression of *Rip2* and NF-κB activation (Supplementary Fig. 5d).

Finally, we performed *in vivo L. monocytogenes* infections in NOD2-tolerized mice in the presence and absence of *Znrf4*. Consistent with the *ex vivo* data, we found that NOD2-tolerized *Znrf4* knockdown mice more effectively controlled *L. monocytogenes* infection as evidenced by lower bacterial loads in both the liver and spleen in comparison with that of control mice (Fig. 7f). The lower bacterial load observed in the spleen of NOD2-tolerized *Znrf4* knockdown mice also correlated with higher levels of IL-6 (Supplementary Fig. 5e). The gene knockdown efficiency in the respective organs was confirmed by immunoblot analysis (Fig. 7f, inset panel). These data demonstrate that ZNRF4 regulates RIP2 levels and is a critical regulatory protein required for NOD2-induced tolerance.

## Discussion

The identification of ZNRF4 as an E3-ubiquitin ligase inducing K48-ubiquitination-mediated degradation of RIP2 provides a novel molecular mechanism for the negative regulation of NOD2 signalling. This is the first report of an E3-ubiquitin ligase that regulates the NOD2 pathway during both acute and NOD2 tolerance conditions through RIP2 degradation.

ZNRF4 belongs to the zinc- and ring-finger (ZNRF) protein family that constitutes four E3-ubiquitin ligase members (ZNRF1, ZNRF2, ZNRF3 and ZNRF4). ZNRF1 has been shown to promote degradation of Akt^32^, whereas ZNRF3 acts as a negative regulator of Wnt signalling pathway by mediating degradation of Wnt receptor components^33^. Thus apart from ZNRF4, other members of ZNRF family are also known to play negative regulatory functions in signalling pathways.

Comparison of our screen results with a previously reported human whole-genome RNAi screen on NOD2 pathway by Warner *et al*.^16^ showed that among the 904 genes identified in our primary screen, 185 genes were common between these two studies (Supplementary Data 1). It is to be noted that while our study used a green fluorescent protein-based NF-κB reporter system in HEK293T cell line, Warner’s study utilized HEK293 cell line with a luciferase-based NF-κB reporter. Studies by our group and that of Warner’s, respectively, identified 17 and 15 genes associated with Crohn’s disease risk are, in fact, regulators of NOD2-NF-κB signalling (Supplementary Data 1); however, only two Crohn’s disease risk genes were common between both studies, highlighting the need for multiple such studies to determine the function of candidate Crohn’s disease susceptibility genes. In addition, comparison of our screen with the previously reported NOD2 druggable genome siRNA screen results of Lipinksi *et al*.^15^ showed that among their 261 hit genes, 10 genes were common with our study (Supplementary Data 1). We also performed meta-analysis of our screen hits with previously published NOD1 siRNA screening studies (please see Supplementary Data 1). Comparison of our screen results with NOD1 screens from other groups showed that 15 genes were common with the druggable siRNA screen by Bielig *et al*.^34^; 22 genes were common with screen by Yeretessian *et al*.^35^ and there were no common genes with the screen by Kim *et al*.^36^ that tested the function of 132 genes in NOD2 signalling. Venn diagram showing the comparison of our screen results with other previously published NOD1/2 screens is also provided as Supplementary Fig. 5f. While several of the known NOD2-NF-κB regulators were discovered, a few regulators were not discovered in our screen possibly due to inherent limitations such as variability in gene knockdown due to inefficient transfection or poorly performing siRNAs. Thus in addition to providing a genome-wide view of NOD1/2 signalling pathway regulation, our study also provide important leads into the mechanism by which Crohn’s disease susceptibility genes contribute to the disease.

Although cIAP1/2 expression was previously implicated in promoting K48-mediated ubiquitination of RIP2, the identity of the E3-ubiquitin ligase that directly K48 ubiquitinates RIP2 remains inconclusive^11^. On the basis of our data, we reason that ZNRF4 binds, K48 ubiquitinates and degrades only a fraction of cellular RIP2 at any given time, to negatively regulate NOD2-mediated signalling and to maintain immune homeostasis. During resting stages, this ensures attenuation of non-desirable basal immune responses induced by excessive RIP2 levels, while maintaining the immune readiness of the cells. It is a well-known fact that increased levels of RIP2 alone can activate NF-κB^29^. The remainder of RIP2 that is unaffected by ZNRF4 is available for pathway activation during NOD2 pathway stimulation. However, we observed an enhanced interaction of RIP2 and ZNRF4 upon NOD2 pathway stimulation. This enhanced interaction at later stages following stimulation subsequently results in progressive attenuation of NOD2 pathway activation, maintaining homeostasis. The transcriptional induction of *ZNRF4* during NOD2 activation is thus an inbuilt cellular mechanism to negatively feedback and control excessive NOD2 signalling and inflammation. Since multiple ubiquitin ligases were previously reported to interact and regulate NOD2 signalling at various stages of activation, an interesting area to investigate further is the potential interplay between various known E3 ligases regulating NOD2 signalling in the context of ZNRF4. Of particular interest would be to examine the interplay of ZNRF4 with ITCH and A20 (ubiquitin-editing complex that deactivates RIP2 following NOD2 pathway activation)^37^, in the context of NOD2 pathway attenuation. The inability of RIP2-K503R RIP2 mutant to trigger efficiently NOD2-dependent NF-κB activation on ectopic expression and the protein mobility shift displayed by the RIP2-K503R mutant protein in comparison with WT RIP2 is notable. Because the K503 of RIP2 targeted by ZNRF4 is located close to the critical autophosphorylation site (Y474) of RIP2 (ref. 38), there may exist a possibility that the post-translational modification on any one of these spatially closer sites of RIP2 could potentially influence the other.

It is noteworthy that we identified the ER as a previously unreported subcellular site of RIP2 localization. We also revealed a novel function for ER as a regulatory platform for NOD2-NF-κB signalling. Consistent with this, recently, it has been reported that ER is involved in the regulation of NF-κB pathway^39^.

Our results also provide a novel mechanistic explanation for how cells develop self-tolerance against chronic MDP stimulation. Previous studies have shown that IRAKM, NF-κB1, ATF3 and TAM receptors also contribute to the attenuation of NOD2 signalling following chronic PRR stimulation in human macrophages^17^,^40^,^41^. Using an unbiased approach, we retrieved ZNRF4 as a new component of NOD2-induced tolerance in human and mouse macrophages. Consistent with the inhibitory function for ZNRF4, MDP-tolerized mice in the absence of ZNRF4 exhibited an enhanced ability to control *L. monocytogenes* infection. Since *ZNRF4* gene silencing abolished NOD2 tolerance in macrophages, we argue that ZNRF4-mediated degradation of RIP2 is a major mechanism behind NOD2 tolerance induction. Given the observation that MDP stimulation (or bacterial stimulation) upregulates ZNRF4 expression (which correlated with reduced RIP2 levels) during tolerance, this constitutes a feedback negative regulatory mechanism to protect cells from excessive inflammation.

Collectively, our study provides critical novel insights into the mechanism by which RIP2 levels are regulated by the cell, leading to the fine-tuning of NOD2 signalling and immune homeostasis. In addition, our results might help to explain (i) how innate immune-tolerant humans are highly susceptible to secondary bacterial infections and (ii) the mechanism by which NOD2 signalling is negatively regulated to maintain immune homeostasis and tolerance. Considering the importance of RIP2 in NOD2 signalling, understanding RIP2 regulation is critical for developing novel therapeutics to modulate NOD2 signalling during infection control and inflammatory conditions. We anticipate that our results will further expand the understanding of the mechanisms behind the pathogenesis arising from perturbations of NOD2 signalling caused by both infections and genetic mutations.

## Methods

### Plasmids and reagents

Human colon epithelial cell line HCT116 (ATCC CCL247), human monocytic cell line THP-1 (TIB-202), human embryonic kidney cells HEK293T and HEK293 (ATCC CRL-3,216, ATCC CRL-1,573) were procured from ATCC. pCDNA 3.1 plasmid expressing RIP2 (HA-tagged) was a gift from Dr G. Nunez (University of Michigan). NF-κB luciferase reporter (E8491) and *Renilla* luciferase (E2241) plasmids were from Promega (Madison, WI, USA). Expression plasmids for human RIP2 (pcDNA-Flag-hRIP2), RIP2ΔCARD (pcDNA-Flag-hRIP2) and RIP2 (pcDNA-Flag-hRIP2CARD) was kindly provided by Dr Maya Saleh (McGill University). Mammalian expression plasmids for Myc-tagged ZNRF4 or -untagged ZNRF4 and its mutant (RING) were obtained from Dr Albert Neutzner (University Hospital Basel). The source and catalogue numbers of other reagents used in this study are: antibodies to RIP2 (sc-22,763, clone number H-300 or sc-166,765, clone number A-10) was procured from Santa Cruz Biotechnology; anti-ZNRF4 antibody was procured from Abcam (ab173954 or ab67951) or from Santa Cruz Biotechnology (sc-169,900) or obtained from Dr Albert Neutzner (University Hospital Basel); phospho-specific antibodies for IκBα (Ser32) (2,859, clone number 14D4), p44/p42 MAPK (extracellular signal-regulated kinase 1/2) (9,106, clone number E10) and p38 MAPK (9,211, Thr180/Tyr18), K48-ubiquitin (4,289), K63-ubiquitin (5,621, clone number D7A11), anti-Myc antibody (2,278, clone number 71D10), anti-rabbit IgG, horseradish peroxidase-linked (7,074) and anti-mouse IgG, horseradish peroxidase-linked (7,076) were procured from Cell signalling Technology; anti-Flag antibody was procured from Sigma-Aldrich (F3165, clone M2); anti-glyceraldehyde 3-phosphate dehydrogenase (GAPDH) antibody was procured from Abcam (ab9482); protein G-beads (SC2002) was from Santa Cruz Biotechnology; CHX (C1988) from Sigma; MDP (tlrl-mdp) and iE-DAP (tlrl-dap) are from Invivogen; Enzyme-Linked Immunosorbent Assay (ELISA) Kits for IL-8 (550,999), IL-1β and TNF (550,610) are from BD Biosciences. Thioglycolate medium was from BD Biosciences (211,716). Transfection reagents used were as follows: DharmaFECT 1 (T-2001-03; Dharmacon), Lipofectamine RNAiMAX (13,778,150; Thermo Fisher Scientific, Carlsbad CA, USA), TransIT-TKO reagent (MIR 2,150; Mirus), Nucleofection Kit (Lonza); QuickChange II XL Site-Directed Mutagenesis Kit (200,521; Agilent Technologies); Dual-Glo Luciferase Assay Kit (E2920; Promega). HEK293 cells stably expressing NF-κB-GFP reporter was from System Biosciences. Cell culture media were purchased from Invitrogen. Luria-Bertani broth was purchased from Sigma. Chemicals such as MG132 and phorbol myristate acetate (PMA) (Calbiochem, San Diego, CA, USA) were reconstituted in dimethylsulfoxide (DMSO). Dual luciferase reagent (Promega) and ECL (Research Instruments) were used in luciferase and for immunoblotting. *In vitro* Ubiquitination Kit was procured from Enzo Lifesciences (BML-UW9920) or Boston Biochem (K-960). *Vivo*-morpholino-targeting ZNRF4 and the *vivo* standard control oligos were purchased from Gene Tools, LLC, USA.

### siRNA transfections and knockdown confirmation

The siRNA was transfected using Dharmafect using lipid DharmaFECT 1 (Dharmacon) or Lipofectamine RNAiMAX (Thermo Fisher Scientific) for HCT116 and HEK293T cells (50 nM) or TransIT-TKO (Miruc Bio) for THP-1 cells (100 nM) or nucleofection strategy (Lonza) for primary human macrophages/CD14+ monocytes (100 nM) according to the manufacturer’s instructions. To verify the knockdown, cells were lysed in the lysis buffer (50 mM Tris (pH 7.5), 150 mM NaCl, 1% Triton X-100, 1 mM EGTA, with protease and phosphatase inhibitors (Pierce Biotech)) for 20 min, and the lysate was separated on 12% denaturing SDS–polyacrylamide gel electrophoresis (SDS–PAGE), followed by immunoblotting using various antibodies. The signal was developed using ECL Western Kit (GE Healthcare) and ImageQuant Imaging System. The details of the siRNAs used are given in Supplementary Table 1. SMARTPool siRNA targeting NOD2 (M-003464-01), NOD1 (M-004398-00), TNFR1 (M-005197-00) and MYD88 (M-004769-01) were purchased from Dharmacon.

### Co-immunoprecipitation assays

For overexpression-based protein–protein interaction studies, Flag-RIP2 (1 μg) or Flag-RIP2ΔCARD (1 μg) or Flag-RIP2CARD (1 μg) with or without Myc-ZNRF4 (100 ng) were transfected into HEK293 cells (700,000 cells per well) for 36 h, treated with MG132 (10 μM) for 2 h and lysed in IP lysis buffer (Cell Signalling Technology) containing 1 mM phenylmethylsulfonyl fluoride, 1 mM sodium orthovandate, 1 mM *N*-ethylmaleimide, 1 × protease/phosphatase inhibitors. For each immunoprecipitation, 0.2 ml (100 μg) of lysates was incubated with 20 μl of G-plus agarose beads (Santa Cruz Biotechnology) for 4 h in a cold room for preclearing. The lysate was incubated with 1 μg of the relevant antibody or control IgG overnight in the cold room, incubated with G-plus agarose beads for 4–6 h, washed three times with lysis buffer and finally the protein complexes were fractionated by SDS–PAGE followed by western blot analysis. For monitoring endogenous protein–protein interaction studies, human primary monocytes (CD14+) or HCT116 cells (250,000 cells per well) were transfected with non-targeting siRNA (si-NT) or ZNRF4 siRNA (si-ZNRF4) (300 or 50 nM, respectively) for 48 h, stimulated with MDP (10 μg ml^−1^) for the desired time points, washed in ice-cold PBS and lysed in IP lysis buffer containing 1 mM phenylmethylsulfonyl fluoride, 1 × protease and phosphatase inhibitors, followed by western blot analysis. For ubiquitination of endogenous RIP2, HCT116 cells were transfected with either NT-siRNA or ZNRF4-siRNA. After 2 days, cells were transfected with HA-ubiquitin. The next day, cells were stimulated with MDP for various time points and lysed in RIPA lysis buffer with 1% SDS (50 mM Tris-HCl, pH 7.8, 150 mM NaCl, 2 mM EDTA, 1% Nonidet P-40, 1% SDS, and protease and phosphatase inhibitor cocktail, 10 mM NEM) lysis buffer. The lysates were denatured by boiling for 10 min, diluted in ten volumes of ice-cold SDS (−) RIPA lysis buffer and immunoprecipitated with anti-RIP2 antibody (2 μg). The immune complexes were washed and eluted with SDS loading buffer.

### *In vitro* RIP2 ubiquitination assays

*In vitro* RIP2 ubiquitination assays were performed using Ubiquitination Kit (Enzo Life Sciences, BML-UW9920 or Boston Biochem, K-960) following the manufacturer’s instructions. The assays were performed in 50 μl reaction volumes with the following components as indicated: 2 μg of each recombinant ubiquitin, recombinant K48, recombinant K48R, recombinant K63, recombinant K63R, 100 nM E1 (Enzo Lifesciences), 2.5 μM E2 (a mixture of E2 enzymes: UbcH1, UbcH2, UbcH3, UbcH5a, UbcH5b, UbcH5c, UbcH6, UbcH7, UbcH8, UbcH10, UbcH13/Mms2) or 100 ng of purified Flag-RIP2 or Myc-ZNRF4 or Myc-ZNRF4ΔRING (purified from transfected HEK293 cells). Following the reaction, the samples were either analysed by immunoblot or denatured and RIP2 was immunoprecipitated. The immune complexes were washed and samples were prepared and probed with anti-K48, anti-K63, anti-total Ubi, anti-Flag or anti-Myc as indicated. The primary antibodies were used at 1:1,000 dilution.

### Subcellular fractionation assay

HCT116 cells were transfected with si-NT or si-ZNRF4 for 48 h, stimulated with MDP (10 μg ml^−1^) for the desired time points. Following MDP stimulation, cytosolic, PM or ER fractions of the cells were extracted. Briefly, cells were washed in ice-cold PBS and the pellet was resuspended in 500 μl of ice-cold 1 × sucrose homogenization buffer (10 mM KCl, 1.5 mM MgCl_2_, 250 mM sucrose, 20 mM HEPES, pH 7.5) containing 1 × protease and phosphatase inhibitors, and cells were lysed by using sonication on ice. Sonicated cells were further passed through syringe (20 G) several times and centrifuged at 500*g* for 10 min, and the supernatant was collected. The supernatant was then centrifuged at 7,000*g* for 10 min to separate the crude microsomal (microsome and cytosol) from the crude mitochondrial fraction, and the crude microsomal fraction (supernatant) was subjected to ultracentrifugation at 100,000*g* for 60 min. The supernatant is a cytosolic fraction. The pellet (PM fraction) was resuspended in the lysis buffer and centrifuged at 6,000*g* for 10 min, and the supernatant was saved. To further enrich for ER compartment, the supernatant was centrifuged at 100,000*g* for 60 min and the pellet was used for the ER fraction. Each compartment fractions were quantitated by Bradford’s assay.

### MDP tolerance induction

THP-1 cells were transfected with 100 nM of si-NT or si-ZNRF4 using TransIT-TKO (Mirus). After 36 h, THP-1 was differentiated into macrophages using PMA for 12 h. The cells were gently washed with PBS and serum-free RPMI medium, and cultured overnight in fresh serum-free RPMI-1640 medium and then processed for various assays. To induce MDP tolerance, the cells were pretreated with MDP (100 μg ml^−1^) for 4 h, gently washed extensively in serum-free medium and maintained in RPMI medium with 10% foetal bovine serum (FBS) for 2–3 h, and gave a second exposure with MDP (100 μg ml^−1^) for 1–8 h (or with *L. monocytogenes* for 4 and 12 h). CD14+ human primary monocytes or mouse peritoneal macrophages were pretreated with MDP (10 μg ml^−1^) for 24 h, gently washed and restimulated with MDP (100 μg ml^−1^) for another 6 or 1 h. The cells were washed with cold PBS and lysed in cell lysis buffer (freshly prepared IX protease inhibitor, 1 mM phenylmethylsulfonyl fluoride, 1 mM sodium orthovandate), incubated on ice for 25 min and centrifuged at 14,000*g* for 15 min. Total protein was quantitated using Bradford’s assay. Equal amounts of total protein were subjected to electrophoresis on 4–20% SDS–PAGE. The membranes were stripped and reprobed with antibodies specific for p-IκBα (1:1,000 dilution), RIP2 (1:1,000 dilution), ZNRF4 (1:500 dilution) or GAPDH (1:5,000 dilution).

### *In vivo* induction of MDP tolerance

C57BL/6J (B6) WT mice were purchased form Biological Resource Center, Agency for Science, Technology and Research (A*STAR) and maintained and housed under specific pathogen-free conditions. All the mice experiments were conducted in accordance with the approved protocols from Institutional Animal Care and Use Committee (IACUC) of A*STAR and Duke-NUS. Age-matched mice aged 7–12 weeks old were used. To elicit peritoneal macrophage recruitment, mice were intraperitoneally injected with 2 ml of 4% thioglycollate medium. After 4 days, mice received 35 mg kg^−1^ body weight MDP intraperitoneally at indicated time points. At 3 h after the second MDP injection, mice were killed and peritoneal cavities were lavaged using 5 ml of ice-cold PBS. Cells were plated in tissue culture petri dishes and allowed to adhere for 2 h at 37 °C. Non-adherent cells were removed and adherent cells (>90% macrophages) were collected by 2 mM EDTA in PBS. Cells were washed and used for *in vitro* stimulation or lysed for western blot analysis. Peritoneal lavage fluid samples were diluted 1:5 and cytokine levels of TNF were measured with ELISA Kits (BD Biosciences).

### Systemic knockdown of *Znrf4* in mice

Translation blocking vMO oligos^31^ targeting *Znrf4* (5′ CATGGCAGTCCCTACCTCCGGCTC-3′) were designed and synthesized by Gene Tools LLC. The *vivo*-morpholino standard control (Gene Tools LLC) was used as the negative control. The next day after thioglycollate injection, 7-week-old C57BL/6J mice were injected with (intravenously, 12.5 mg kg^−1^ body weight) vMO for three consecutive days followed by the MDP treatment on day 4. Mice received 35 mg kg^−1^ body weight MDP (intraperitoneally) at indicated time points. At 3 h after the second MDP injection, mice were killed and various organs were collected, lysed and analysed for the expression of various proteins by western blot analysis.

### Cell culture and bacterial infection experiments

THP-1 cells (cultured in RPMI-1640 medium with 10% FBS) at 37 °C was treated with 100 nM PMA for 12 h to differentiate as macrophages. The cells were washed with serum-free media and cultured overnight in fresh serum-free medium and then processed for various assays. Human primary monocytes were purified from human peripheral blood mononuclear cell by positive CD14 selection. Monocytes were cultured for 7 days with 10 ng ml^−1^ macrophage colony-stimulating factor for differentiation to macrophages. For MDP tolerance studies, CD14+ monocytes (lineage-HLA-DR+CD14+CD16low) were purified from human peripheral blood mononuclear cell by FACS analysis. Human colon epithelial cell HCT116 was maintained in Dulbecco’s modified Eagle’s medium supplemented with 10% FBS media.

Before infection with *L. monocytogenes*, the cells were washed gently with PBS. Overnight grown bacteria were collected by centrifugation (3,000*g* for 8 min), washed two times with PBS and resuspended in respective media before infection. Infections were performed at different multiplicity of infections (MOIs). To synchronize entry, the bacteria were centrifuged at 600*g* for 5 min. Following 1 h incubation with bacteria, the cells were washed three times with PBS and treated with media containing 100 μg ml^−1^ gentamicin for another 1 h to kill extracellular bacteria. Subsequently, the cells were washed three times with PBS and either lysed in PBS containing 0.1% Triton X-100 and plated on Luria-Bertani agar plates to assess invasion (by calculating the colony-forming units (CFU)) denoted as 0 h or maintained in media with 10 μg ml^−1^ gentamicin for various time points.

### Infection of mouse peritoneal macrophages *ex vivo*

Following MDP tolerance induction in mice, the peritoneal cavities were lavaged using 5 ml of ice-cold PBS. The cells were resuspended in RPMI-1640 media supplemented with 10% FBS to a final concentration of 6 × 10^5^ cells per ml and plated in 24-well plates. Macrophage viability was determined by Trypan blue exclusion, and was determined to be >95%. After 4–6 h, the cells were infected with *L. monocytogenes* (MOI=1). After 2 h, the cells were gently washed four times with warm PBS. To determine the invasion rate in Control vMo or ZNRF4 vMo, the cells were lysed in PBS containing 0.1% Triton X-100, and plated on nutrient agar plate (this time point is indicated as 2 h). Rest of the cells (MDP 3 h, MDP6h+3 h) was incubated in RPMI-FBS medium containing gentamicin (50 μg ml^−1^) for 1 h to kill extracellular bacteria. After washing four times with warm PBS, the cells were incubated further in RPMI-FBS medium without gentamicin. At 24 h after infection, the peritoneal macrophages were lysed in 1 × PBS containing 0.1% Triton X-100, and plated on nutrient agar plates. The number of CFU per ml was determined after 12–18 h of incubation at 37 °C, with each dilutions plated in triplicate.

### *In vivo* infection of mice with *L. monocytogenes*

The mice were divided into three groups for treatment: (1) control vMo/ZNRF4 vMo PBS only; (2) control vMo/ZNRF4 vMo MDP 3 h; (3) control vMo/ZNRF4 vMo MDP 6+3 h. For *in vivo* infection, these mice groups were infected intraperitoneally with *L. monocytogenes* (4 × 10^4^ bacteria per mouse). At 24 h after infection, mice were killed and peritoneal cavities were lavaged using 5 ml of ice-cold PBS. Cell lysates were analysed for ZNRF4, RIP2 and p-IκBα levels by western blot. From the same mice, liver and spleen were collected, and lysed in 1 × PBS buffer. The liver and spleen were homogenized in 1-ml PBS and placed on ice. Dilution of 100 μl liver or spleen tissue homogenate was mixed with 900 μl PBS. Four serial 10-fold dilutions in PBS were prepared and plated on nutrient agar plates, and incubated for 12–18 h at 37 °C, with each dilution plated in triplicate. The colonies were then counted and viable bacteria were expressed in CFU per organ.

### Purification of recombinant proteins

HEK293 cells were transfected with 10 μg of Flag-RIP2 or Myc-ZNRF4 or Myc-ZNRF4ΔRING separately. After 36 h, the cells were washed two times in ice-cold PBS, and lysed in 1 × cell lysis buffer containing protease inhibitors. Flag-RIP2 lysate was incubated with 3 × Flag beads (20 μl) overnight. Similarly, Myc-ZNRF4 and Myc-ZNRF4ΔRING lysates were incubated with 2 μg of Myc antibody. The following day, the beads were extensively washed with the lysis buffer containing protease inhibitors. The protein bound to beads was eluted with the respective peptide (Flag or Myc), and the beads were centrifuged and the supernatant containing respective proteins were stored at −80 °C before *in vitro* ubiquitination assays were performed. The total quantity of protein was estimated by Bradford’s assay.

### RIP2 degradation assay

The 293 cells (300,000 cells per well) were grown in 24-well plate overnight, and transfected with Flag-RIP2/RIP2 mutants (250 ng) or empty vector with or without Myc-tagged or -untagged ZNRF4 (25 ng) or Myc-ZNRF4ΔRING (25 ng), respectively. After 24 h, the cells were treated with DMSO alone or MG132 (10 μM) for 2 h. The cells were lysed in western cell lysis buffer containing 10 mM of NEM and protease/phosphatase inhibitors, and the degradation of RIP2 profile was monitored by SDS–PAGE using antibodies targeting tags Flag (Sigma) or Myc (Roche) or ZNRF4 antibody, and GAPDH as a loading control respectively.

### Luciferase reporter gene assays

HEK293T cells stably expressing NOD2 (250,000 cells per well) in 24-well dishes were transfected with NF-κB luciferase reporter plasmid, internal control pTK-RL plasmid constitutively expressing *Renilla* luciferase (Promega), gene plasmids or control empty plasmids (Flag- or Myc- or HA-tagged) using the lipid Fugene (Roche). Following stimulation with MDP for 8 or 24 h, the luciferase activity was determined using the Dual-Glo Assay Kit (Promega). Firefly luciferase activities were normalized using *Renilla* luciferase activities.

### Quantitative RT–PCR analysis assay

Total RNA was isolated using RNAeasy Kit (Qiagen) and cDNA was made using iSCRIPT Kit (Bio-Rad) according to the manufacturer’s instructions. mRNA levels were determined by SYBR Green dye-based quantitative real-time PCR (qRT–PCR), with primers detecting ZNRF4 or RIP2 or mouse *Il-6* and normalized with human β-actin gene/mouse *GAPDH*. The primer information is given in Supplementary Table 1. Results were analysed using delta-delta-Ct algorithm.

### Quantitation of cytokines and chemokine production by ELISA

The cell culture supernatants were collected at various time points treated or untreated with the indicated ligands were stored at −80 °C until ELISA was done. The protein standards and antibody pairs for IL-8, IL-1β and TNF were procured from BD Bioscience. The mouse ELISA Kit for TNF and IL-6 was obtained from eBiosciences. ELISA experiments for cytokine measurement were performed according to the manufacturer’s instructions.

### Immunofluorescence microscopy experiment

Colocalization of various markers was performed using confocal microscopy. For triple staining, HCT116 cells were grown on coverslips and transfected with various plasmids (0.3 μg each)—pmTurquoise2-ER, YFP-ZNRF4 and HA-tagged RIP2. After 24 h, the cells were fixed with 4% paraformaldehyde, permeabilized and were stained with anti-HA antibody (AB71113).

For double staining of ER and RIP2, HCT116 cells were transfected with mCherry-Sec61β and HA-RIP2 plasmid (0.5 μg each). Cells were subsequently fixed, permeabilized and stained with anti-RIP2 antibody (sc22763), followed by a secondary Alexa488 antibody. For double staining of ER and ZNRF4, HCT116 cells were transfected with mCherry-Sec61β and ZNRF4 plasmid (0.5 μg each). Cells were then stained with fixed, permeabilized and stained with anti-ZNRF4 antibody (sc169900), followed by a secondary Alexa488 antibody. The coverslips were mounted using Prolong Gold Antifade (Molecular Probes) and examined using Zeiss confocal microscope with appropriate filter sets. All the images were acquired on LSM 710 Carl Zeiss microscope using Plan-Apochromat × 63/1.40 oil DIC objective. Images were acquired at 16 bit depth at a resolution of 1,024 × 1,024 pixels. Images were analysed using the Zen 2010 Software and were processed using the Adobe Photoshop7 Software. Quantification of the colocalization of various proteins was done using the Zen 2010 Software (Zeiss), which calculates the Mander’s overlap coefficient^42^. The values for the overlap coefficient range from 0 to 1. An overlap coefficient with a value of 1 indicates complete colocalization and 0 represents zero colocalization. The overlap coefficients were calculated using data from at least three independent experiments (*n*≥50).

### Rescue experiment

ZNRF4 was knockdown in HCT116 cells using siRNA (pool) targeting the 3′-UTR. 36 h following the knockdown, cells were transfected with plasmids encoding WT or RING mutant of ZNRF4 along with NF-κB and *Renilla* luciferase plasmids followed by MDP stimulation and luciferase assay.

### NF-κB p65 transcription factor assay

HCT116 cells were treated with ZNRF4 siRNA or NT siRNA for 48 h followed by MDP stimulation for various time points. At each time point, cells were collected, nuclear extract was prepared and the DNA-binding activity of NF-κB/p65 was assessed using NF-κB p65 Transcription Factor Assay Kit (Abcam; ab133112) according to the manufacturer’s instructions.

### CHX experiment

Human primary monocytes (CD14+) were treated with either CHX alone (25 μg ml^−1^) or with MDP and CHX together for 0, 4 and 8 h. Lysates were prepared and immunoblotting was performed.

### RNAi screen methodology

The RNAi screen used a fluorescence microscopy-based assay in HEK293T cell line stably expressing both human NOD2 and an NF-κB target promoter-driven GFP reporter (293T-NOD2-NF-κB-GFP), with MDP as the ligand. The unstimulated 293T-NOD2-NF-κB-GFP cells showed only negligible significant background signal; stimulation with MDP for 24 h led to a robust increase in the number of GFP+ cells. Silencing of both RIP2 and NOD2, but not of MYD88, NOD1 and TNFR1, reduced GFP signal drastically (Supplementary Fig. 1a); this proved that the observed increase in GFP+ cells is exclusively due to signalling through NOD2-RIP2 axis. RNAi screen was performed using a condition where stimulation with MDP (10 μg ml^−1^) for 24 h would lead to ∼30% of the 293T-NOD2-NF-κB-GFP cells becoming GFP+; this would enable efficient discovery of both positive and negative regulators. The screen was performed in 384-well format in duplicates, and 50 nM siRNA was transfected into each well of 6,000 cells for 48 h, using the lipid DharmaFECT 1, before MDP stimulation. The activation level of the NF-κB-GFP reporter was imaged using high content microscopy (× 4 magnification; ImageXpress Micro, Molecular Devices Corporation), and expressed as the per cent of GFP+ cells per well (cells were defined as 4,6-diamidino-2-phenylindole-stained nucleus). The algorithm MetaXpress (version 3, Molecular Devices Corporation) was used for data quantitation. The entire RNAi screen was conducted in a bipartite manner. First, a primary screen targeting each gene with a pool of four siRNAs (targeting unique regions of the target) was conducted. Subsequently, we conducted a secondary screen in which each siRNA in the pool was tested individually to minimize the number of off-target hits, followed by a tertiary screen to filter out the general NF-κB regulators (counterscreen for NF-κB activation through TLR3). The *Z*-score normalization was performed by subtracting the mean of the values of the corresponding 384-well plate from the values for each gene silencing and dividing by the standard deviation of the plate. Genes were scored as final ‘hits’ only if knockdown with at least two independent siRNAs altered the signal by a *Z*-score of ±2.5 for positive and negative regulators, respectively.

### Molecular concept mapping for functional enrichment

To assess the statistical enrichment of functional gene sets from molecular function, biological process categories and pathways for candidate genes, the *P* value of functional enrichment was determined using the hypergeometric test^43^. The gene sets were compiled from Gene Ontology^44^, KEGG, BioCarta and Reactome. For the functional enrichment map, the enrichment results were represented as a network graph, with nodes denoting enriched gene sets or categories as described previously^45^,^46^,^47^. Node size corresponds to the number of candidate genes in each gene set or category, while the node colour intensity corresponds to the gene set enrichment score (−log_10_(*P* value)). Thickness and colour intensity of edges denote the extent of mutually overlapping genes between gene sets. The Jaccard and overlap coefficients were used to compute the overlap between gene sets^48^. Strongly connected components in the network were identified using Tarjan’s algorithm^49^.

### Protein interaction data

Protein–protein interaction data was obtained from the Human Protein Reference Database^50^. Network construction uses a graph theoretic representation in which nodes denote components (gene products) and edges protein–protein interactions as described previously^51^. Briefly, these incorporate graph layout descriptions in the Dot language^52^, which implements an iterative solver (Newton–Raphson algorithm) that searches for low-energy configurations and creates a virtual physical model (Spring model)^53^ to optimize the graph layout for visualization. Known ubiquitination-related genes (Supplementary Fig. 2b) were selected as described^54^.

### Protein elution and in-gel tryptic digestion

For mass spectrometric analysis, 293T cells were first transfected with plasmid expressing NOD2, RIP2, using polyethylenimine. At 24 h after transfection, cells were incubated with media containing 25 μM MG132 for 4 h, followed by 1 h of 10 μg ml^−1^ MDP stimulation. Cells were then lysed and immunoprecipitation was performed using mouse anti-Flag antibody (Sigma). Following immunoprecipitation, the beads used to pull down the ubiquitinated proteins were boiled in gel loading buffers. The eluted samples were run on an SDS–PAGE gel. Gel slices were cut into small pieces and transferred to Eppendorf tubes. They were washed several times with Milli-Q water, and then followed with 50% acetonitrile (ACN)/50% 25 mM NH_4_HCO_3_ via vigorous vortexing for 30 min. The gel pieces were then dehydrated with 100% ACN. They were then reduced with 10 mM dithiothreitol at 56 °C for 1 h and alkylated with 55 mM iodoacetamide for 45 min in the dark followed by successive washes with 25 mM NH_4_HCO_3_ and 50% ACN/50% 25 mM NH_4_HCO_3_ with vigorous vortexing for 30 min. The gel pieces were dehydrated again with 100% ACN. Trypsin (V5111; Promega) was added in the ratio of 1:50. After the trypsin solution was completely absorbed by gel particles, 25 mM NH_4_HCO_3_ was added to completely cover the particles. They were then incubated at 37 °C overnight. Peptides were extracted from gel particles with 50% ACN containing 0.1% trifluoroacetic acid under sonication for 30 min two times. The combined extracts were dried in vacuum and stored at −20 °C before liquid chromatography tandem-mass spectrometry (LC-MS/MS) analysis.

### LC-MS/MS analysis

Tryptic peptides were dissolved in 0.1% formic acid (FA) in 2% ACN. They were then analysed on a Dionex Ultimate 3,000 RSLCnano system coupled to a Q Exactive tandem mass spectrometer (Thermo Fisher Scientific). Each peptide sample was injected into an Acclaim peptide trap column (Thermo Fisher) via the autosampler of the Dionex RSLCnano system. Peptides eluted from the peptide trap were separated in a Dionex EASY-spray column (PepMap C18, 3 μm, 100 A, 75 μm × 15 cm) (Thermo Fisher Scientific) at 35 °C. Mobile phase A (0.1% FA in H_2_O) and mobile phase B (0.1% FA in 100% ACN) were used to establish a 60 min gradient at a flow rate of 300 nl min^−1^. Peptides were then analysed on Q Exactive with an EASY nanospray source (Thermo Fisher Scientific, Waltham MA, USA) at an electrospray potential of 1.5 kV. A full MS scan (350–1,600 *m*/*z* range) was acquired at a resolution of 70,000 at an *m*/*z* of 200 and a maximum ion accumulation time of 100 ms. Dynamic exclusion was set as 30 s. Resolution for HCD spectra was set to 35,000 at an *m*/*z* of 200. The AGC setting of full MS scan and MS2 were set as 1E6 and 2E5, respectively. The 10 most intense ions above a 1,000 counts threshold were selected for HCD fragmentation with a maximum ion accumulation time of 120 ms. Isolation width of 2 Th was used for MS2. Single and unassigned charged ions were excluded from MS/MS. For HCD, normalized collision energy was set to 28%. The underfill ratio was defined as 0.1%.

### LC-MS/MS data analysis

Briefly, raw data files were converted to the mascot generic file format using Proteome Discoverer version 1.4 (Thermo Electron, Bremen, Germany) with the MS2 spectrum processor for deisotoping the MS/MS spectra. The concatenated target-decoy Uniprot human database (sequence 88,473, downloaded on 29 November 2013) was used for MS/MS spectra searches. The database search was performed using an in-house Mascot server (version 2.4.1; Matrix Science, Boston, MA, USA) with MS tolerance of 10 p.p.m. and MS/MS tolerance of 0.02 Da. Two missed trypsin cleavage sites per peptide were tolerated. Carbamidomethylation (C) was set as a fixed modification, while oxidation (M), deamidation (N and Q) and ubiquitination (GG at K) were variable modifications. Annotated MS/MS spectra of Mascot-detected ubiquitinated peptides were exported in the Mascot Peptide View format and shown in Supplementary Data (Supplementary Data 2; annotated MS/MS spectra in Mascot Peptide View).

### Statistical analysis

All data were analysed by an unpaired two-tailed Student’s *t*-test or one-way analysis of variance. Values of *P*≤0.05 were considered statistically significant.

### Data availability

The authors declare that the data supporting the findings of this study are available within the article and its Supplementary Information Files, or are available from the corresponding authors on request.

## Additional information

**How to cite this article:** Bist, P. *et al*. E3 Ubiquitin ligase ZNRF4 negatively regulates NOD2 signalling and induces tolerance to MDP. *Nat. Commun.* **8**, 15865 doi: 10.1038/ncomms15865 (2017).

**Publisher’s note:** Springer Nature remains neutral with regard to jurisdictional claims in published maps and institutional affiliations.

## Supplementary Material

Supplementary Information

Supplementary Data 1

Supplementary Data 2
