#  Erratum: E3 Ubiquitin ligase ZNRF4 negatively regulates NOD2 signalling and induces tolerance to MDP

**DOI:** 10.1038/ncomms16141

**Published:** 2017-08-18

**Authors:** Pradeep Bist, Wan Shoo Cheong, Aylwin Ng, Neha Dikshit, Bae Hoon Kim, Niyas Kudukkil Pulloor, Hanif Javanmard Khameneh, Matija Hedl, Avinash R. Shenoy, Vanniarajan Balamuralidhar, Najib Bin Abdul Malik, Michelle Hong, Albert Neutzner, Keh-Chuang Chin, Koichi S. Kobayashi, Antonio Bertoletti, Alessandra Mortellaro, Clara Abraham, John D. MacMicking, Ramnik J. Xavier, Bindu Sukumaran

Nature Communications 8 Article number: 15865 ; DOI: 10.1038/ncomms15865 (2017); Published: 06 28 2017; Updated: 08 18 2017

The previously published version of this Article contained a line number in the Abstract within the sentence ‘A fraction of RIP2 localizes to the endoplasmic reticulum (ER), where it interacts with ZNRF4 under either unstimulated and muramyl dipeptide-stimulated conditions’. This has now been corrected in the PDF and HTML versions of the Article.

