# Supplementary material for: E3 Ubiquitin ligase ZNRF4 negatively regulates NOD2 signalling and induces tolerance to MDP
Source: Nat Commun. 2017 Jun 28;8:15865. doi: 10.1038/ncomms15865 (PMC5493756; doi:10.1038/ncomms15865)
Supplement: Supplementary Information [file ncomms15865-s1.pdf]

Description of Supplementary Files

File name: Supplementary Information
Description: Supplementary figures and supplementary table.

File name: Supplementary Data 1
Description: siRNA screen results

File name: Supplementary Data 2
Description: Peptide sequences with ubiquitinated lysine 503 site (red) identified

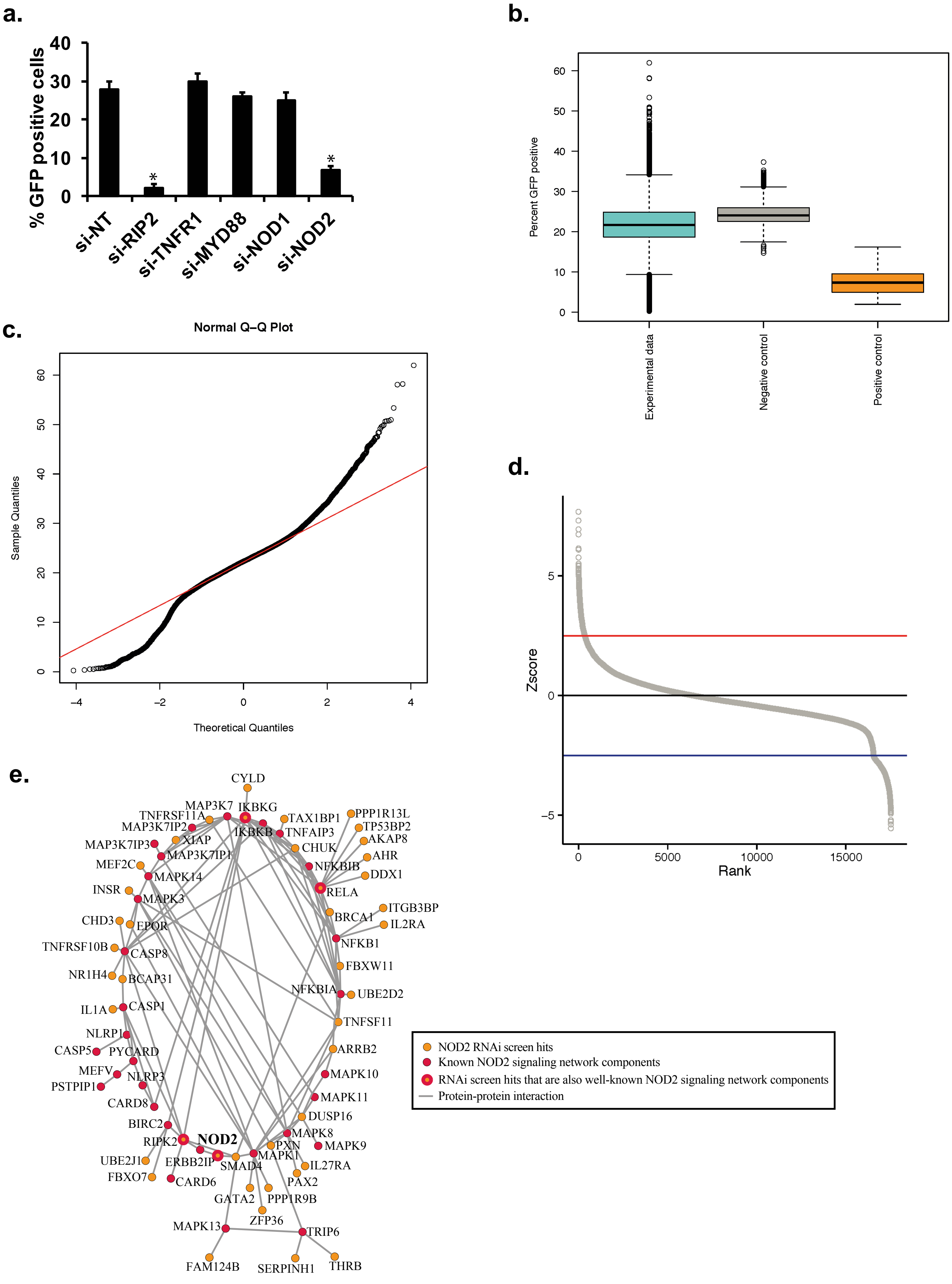

Supplementary Figure 1. RNAi screen assay optimization and data statistical analysis. (a) Validation of NF-κB reporter assay. 293T-NOD2-NF-κB-GFP cells

silenced for RIP2, NOD1, NOD2, MYD88 and TNFR1 were stimulated with MDP, and the percent GFP positive cell were determined at 24h by fluorescence microscopy. Graphs show mean ± SD from three independent experiments, * $p<0.05$ (Student's *t* test). Significance is expressed by comparing to the values obtained from si-NT treated samples. **(b), (c) and (d) Statistical summary plots of the siRNA screen data. (b)** Distribution of the signal intensity quantified by the percentage of GFP-positive cells plotted for the screen data (cyan), the negative control (grey), and positive control (orange). **(c)** Q-Q plot of percentage GFP-positive cells against a normal distribution. **(d)** Plot showing the Z-score distribution among 18,120 gene silencing events. The red line indicates the threshold (+2.5) for candidate negative regulators and the blue line indicates the threshold (-2.5) for putative positive regulators of NOD2-mediated NF-κB signaling. **(e)** A network showing the screen hits that interacts (through protein interactions) with known components in the NOD2 signaling network.

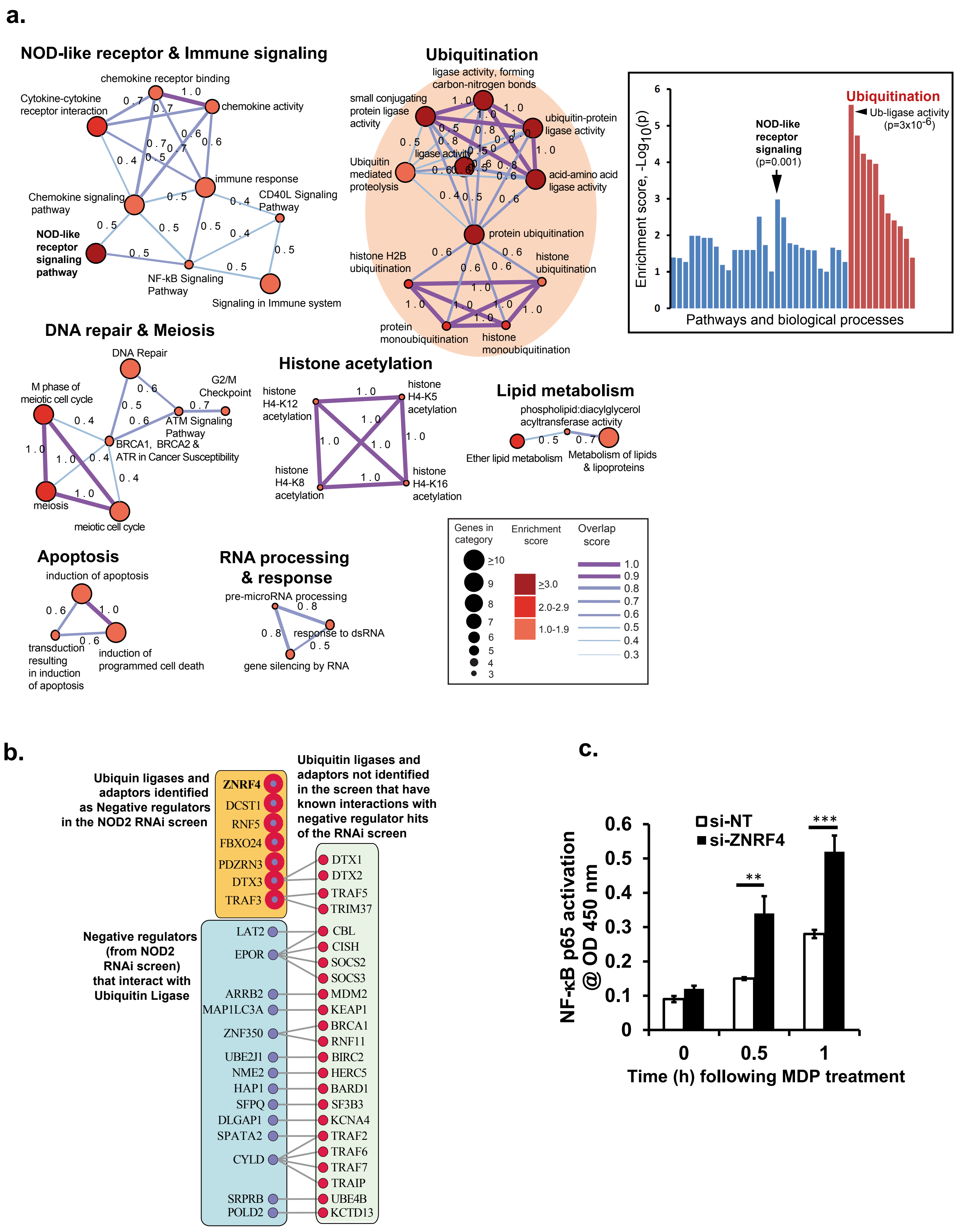

Supplementary Figure 2. (a and b) Enrichment analysis of RNAi screen results. (a) Map of molecular concepts representing enriched pathways and biological processes

amongst 904 primary RNAi screen hit genes. This map displays mutual overlap between gene sets of biological pathways and processes, and then clusters highly connected gene sets that are enriched among the screen hits into ‘molecular concepts’. Nodes represent enriched gene sets while edges denote the extent of mutually overlapping hit genes between gene sets, as detailed in the graphical legend (boxed). Processes associated with ubiquitination were the most over-represented. **(b)** Newly identified ubiquitination-related negative regulators of NOD2 signaling. Novel negative regulators that are ubiquitin ligases and their adaptors are highlighted in the yellow box. Negative regulators (blue box) that potentially interact with known ubiquitination-related genes (green box) through first-order (direct) protein-protein interactions (grey edges) are shown. **(c) DNA binding activity of NF-κB p65 is increased in *ZNRF4*-silenced HCT116 cells upon MDP stimulation.** HCT116 cells were transfected with transfected with either NT-siRNA or ZNRF4- siRNA and stimulated with MDP for various time points. Nuclear extracts were prepared and DNA binding activity of NF-κB p65 was calculated by measuring the OD @ 450nm. ** $p<0.01$, *** $p<0.001$ (Student’s *t* test). Data represents the mean ± SD of triplicate samples and are representative of three independent experiments. si, siRNA; si-NT, negative control siRNA.

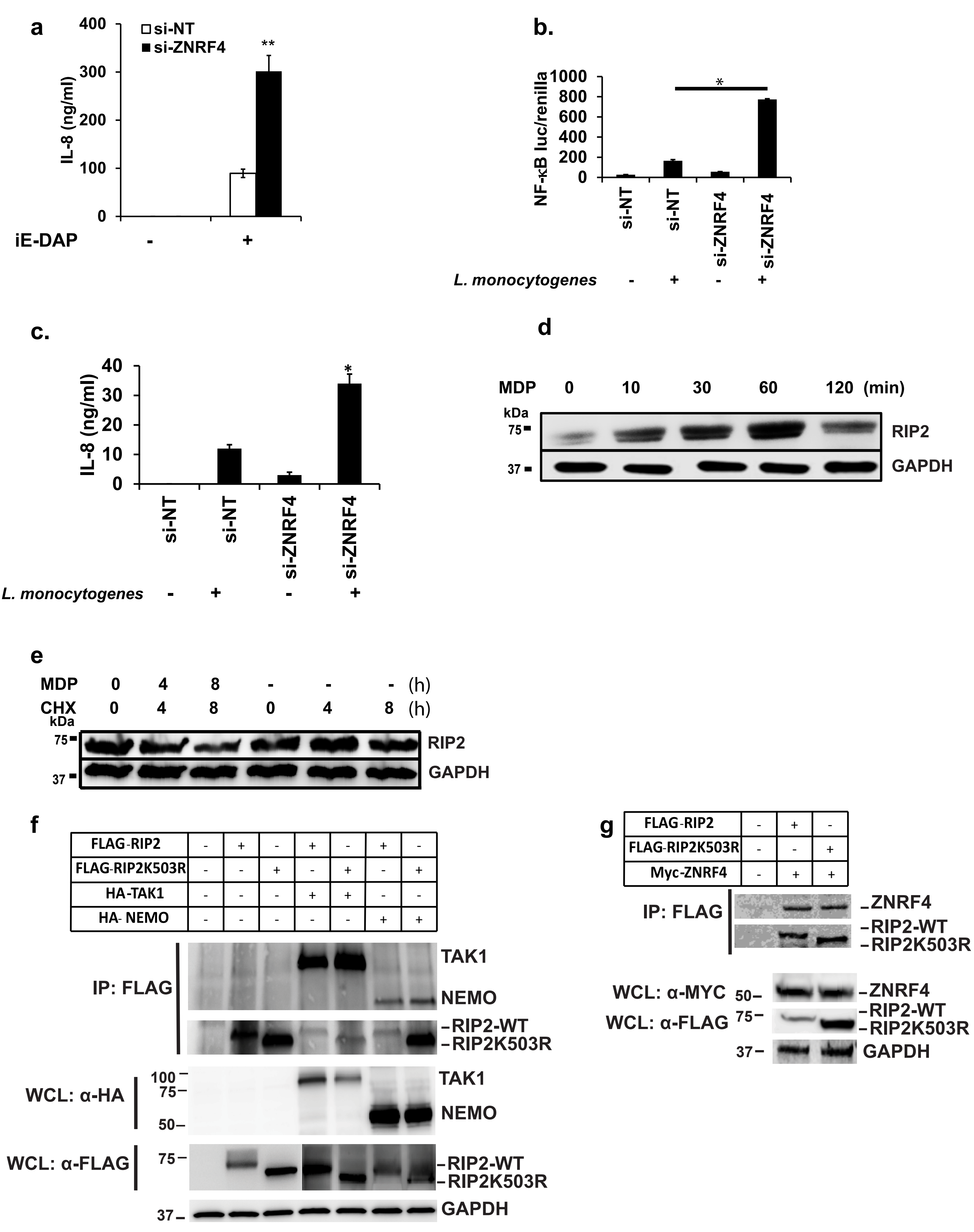

Supplementary Figure 3: **ZNRF4 expression attenuates NOD1/NOD2-mediated innate immune response. (a)** IL-8 secretion in ZNRF4 knock down human primary macrophages following iE-DAP treatment. **(b)** NF-κB luciferase activity in HCT116 cells

transfected with si-NT, or si-ZNRF4 and stimulated with *L. monocytogenes*, normalized with internal control Renilla luciferase activity **(c)** Enzyme-linked immunosorbent assay of IL-8 in the culture supernatants of HCT116 cells transfected with si-NT, or si-ZNRF4 and stimulated with *L. monocytogenes*. **(d and e) MDP treatment induces RIP2 degradation in human primary monocytes. (d)** Immunoblot to measure the protein expression levels of RIP2 and pIκB in human primary monocytes (CD14+) following MDP stimulation. **(e)** Immunoblot to measure the protein expression levels of RIP2 in human primary monocytes (CD14+) following treatment with either cycloheximide (CHX) alone or with MDP and CHX together for 0, 4 and 8 h. Data in **a**, **b**, and **c** represents the mean ± SD of triplicate samples and are representative of three independent experiments. * $p<0.05$, ** $p<0.01$ (Student's *t* test). si, siRNA; si-NT, negative control siRNA. **(f and g) K503R mutant of RIP2 interacts with NOD2 signalling pathway components. (f)** FLAG-tagged wild type RIP2 (RIP2-WT) or K503R mutant of RIP2 (RIP2K503R) was co-expressed with **(f)** HA-tagged TAK1, NEMO or **(g)** myc-tagged ZNRF4 and immunoprecipitated with FLAG antibody and immunoblotted with various proteins. The Western blot is representative of one of three independent experiments that were conducted.

a.

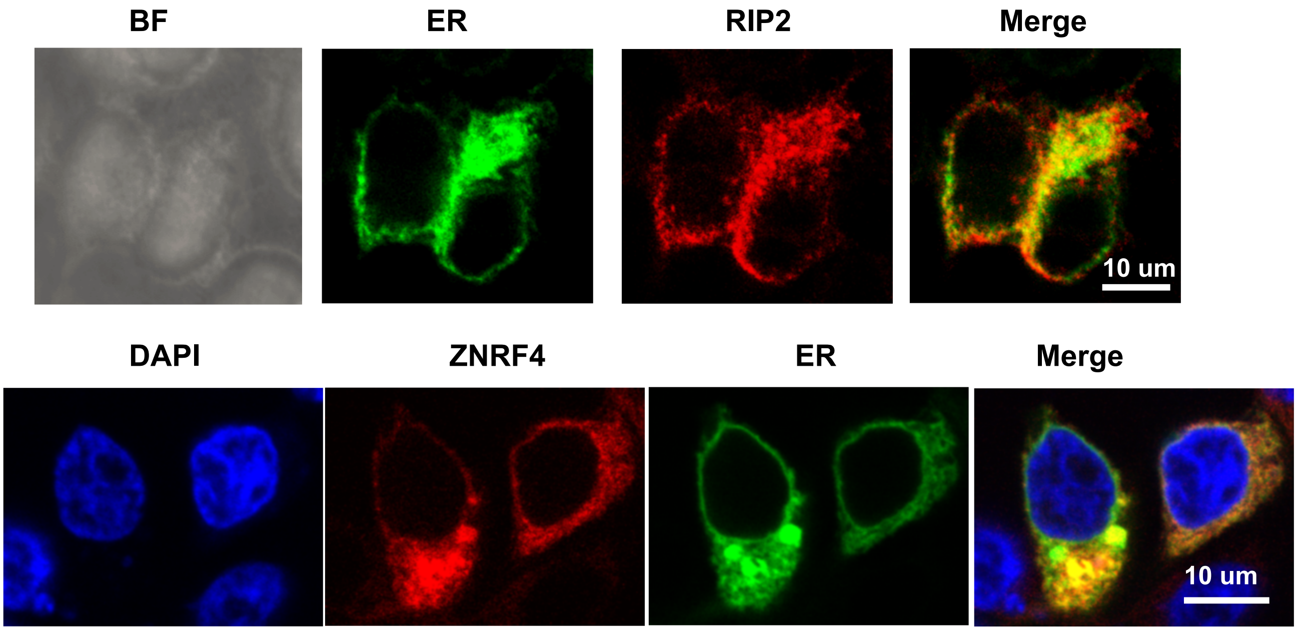

b.

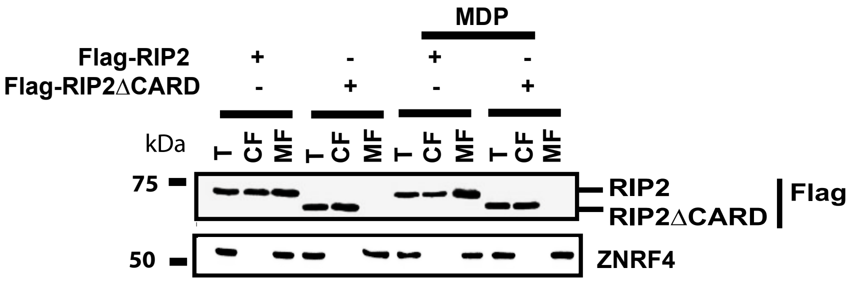

Supplementary Figure 4. Localization patterns of ZNRF4, wild-type and mutant RIP2. a (top row) RIP2 colocalizes with ER. HCT116 cells were transfected with mcherry sec61B and HA RIP2 plasmid. Cells were fixed, permeabilised and stained for RIP2. Cells were analyzed for the colocalizartion between ER (green) and RIP2 (red) by confocal microscopy. Images were acquired at 63X optical magnification. Scale bars denote 10µm. **a (bottom row) ZNRF4 colocalizes with ER**. HCT116 cells were transfected with mcherry sec61B and ZNRF4 plasmid. Cells were fixed, permeabilised and stained for ZNRF4. Cells were analyzed for the colocalizartion between ER (green) and ZNRF4 (red) by confocal microscopy. Images were acquired at 63X optical magnification. Scale bars denote 10µm. Confocal images are representative of one of three independent experiments that were conducted. **(b)** Immunoblot analysis for RIP2 from various cellular fractions of HCT116 cells transfected with expression plasmid encoding Flag-RIP2 or Flag-RIP2ΔCARD and stimulated with MDP. Total (T), cytosolic (CF), membrane (MF) fractions. Equal amount of each fraction (30 µg) was analyzed by Western blot. The Western blot is representative of one of three independent experiments that were conducted. BF=Bright Field.

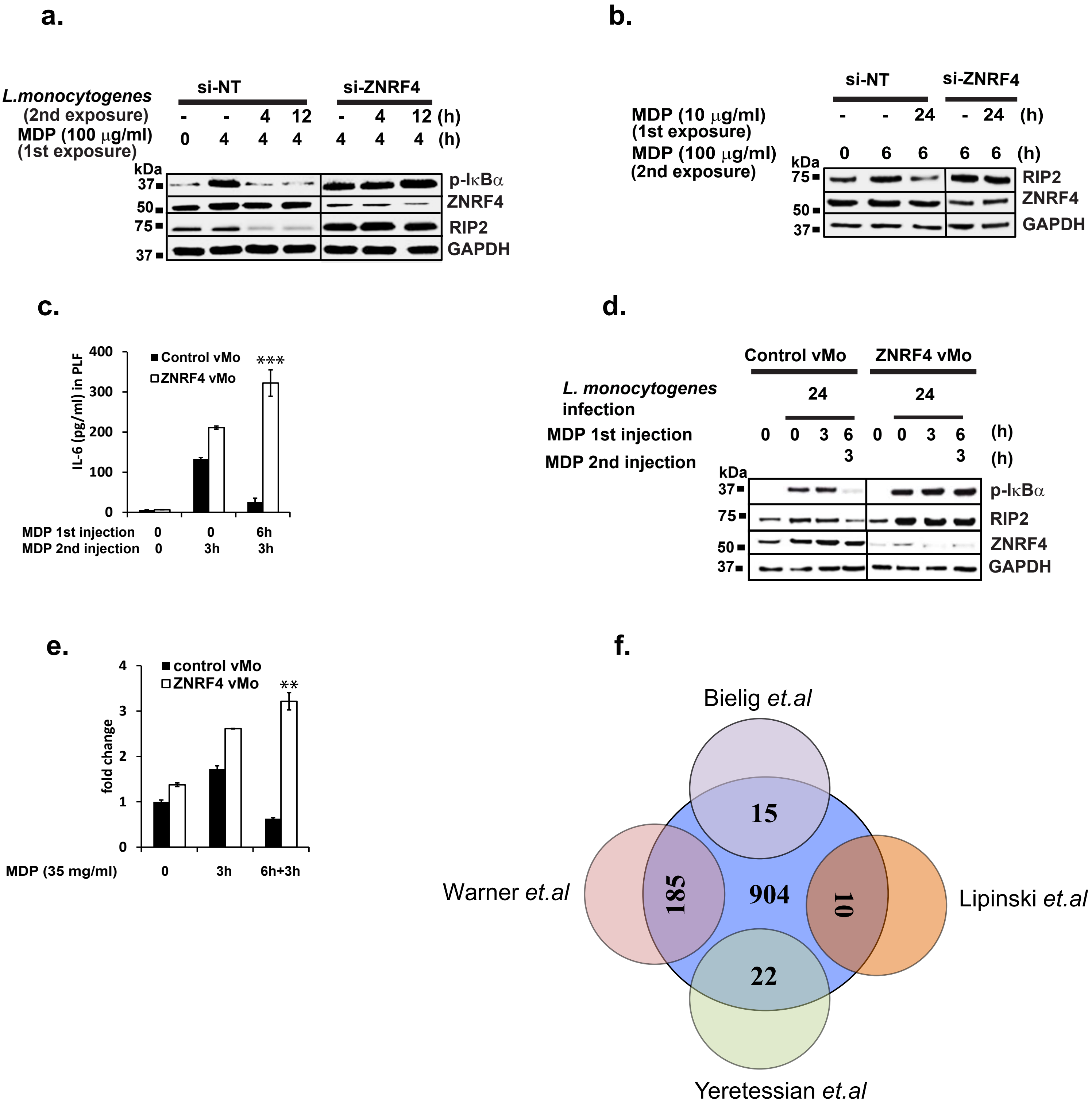

Supplementary Figure 5. ZNRF4 is critical for the induction of MDP-tolerance. **(a)** Immunoblot analysis of THP-1 cells transfected with *ZNRF4*-specific siRNA (si-ZNRF4) or non-targeting siRNA (si-NT), and pre-treated with MDP for 4 h, washed and re-stimulated with *L. monocytogenes* for the indicated time intervals. **(b)** Immunoblot analysis of *ZNRF4*-siRNA (si-ZNRF4) or non-targeting siRNA (si-NT) treated human primary monocytes ($CD14^{+}$) under non-tolerant or MDP-tolerant conditions. To induce MDP-tolerance, cells received MDP pre-treatment, washed and re-stimulated with MDP

for the indicated time intervals. **(c)** Thioglycollate-treated mice received intravenously either vivo-morpholino targeting murine ZNRF4 (ZNRF4 vMO) or vivo-morpholino standard control (Control vMO), followed by the MDP treatment on day four for the indicated time points, and ELISA for IL-6 in peritoneal lavage fluid was performed. **(d)** Thioglycollate-treated mice received intravenously ZNRF4 vMO or Control vMO. On fourth day, MDP or endotoxin free PBS was injected *i.p* for the indicated time points. After the last injection of MDP, peritoneal lavage was harvested, and infected *ex-vivo* with *L. monocytogenes (m.o.i=1)*. Cell lysates were subjected to Western blot analysis for p-IκBα, ZNRF4, RIP2, and GAPDH respectively. Western blots are representative of one of three independent experiments that were conducted. **(e)** Comparison of the levels of IL-6 from the mouse spleens of *L. monocytogenes*-infected and NOD2-tolerized *Znrf4* knock down mice by qRT-PCR. The results are represented as the fold-change of IL-6 in each group compared to the control (control-vMo-0h). Data in **c** and **e** are representative of two independent experiments with similar results and represents mean ± SD. ** p<0.01, *** p<0.001 (Student's t test). **(f)** Venn diagram showing the overlap between the hit genes identified in the current study and those identified in the four published RNAi screens on NOD1/2 pathway. Central blue circle lists hits from the current study.

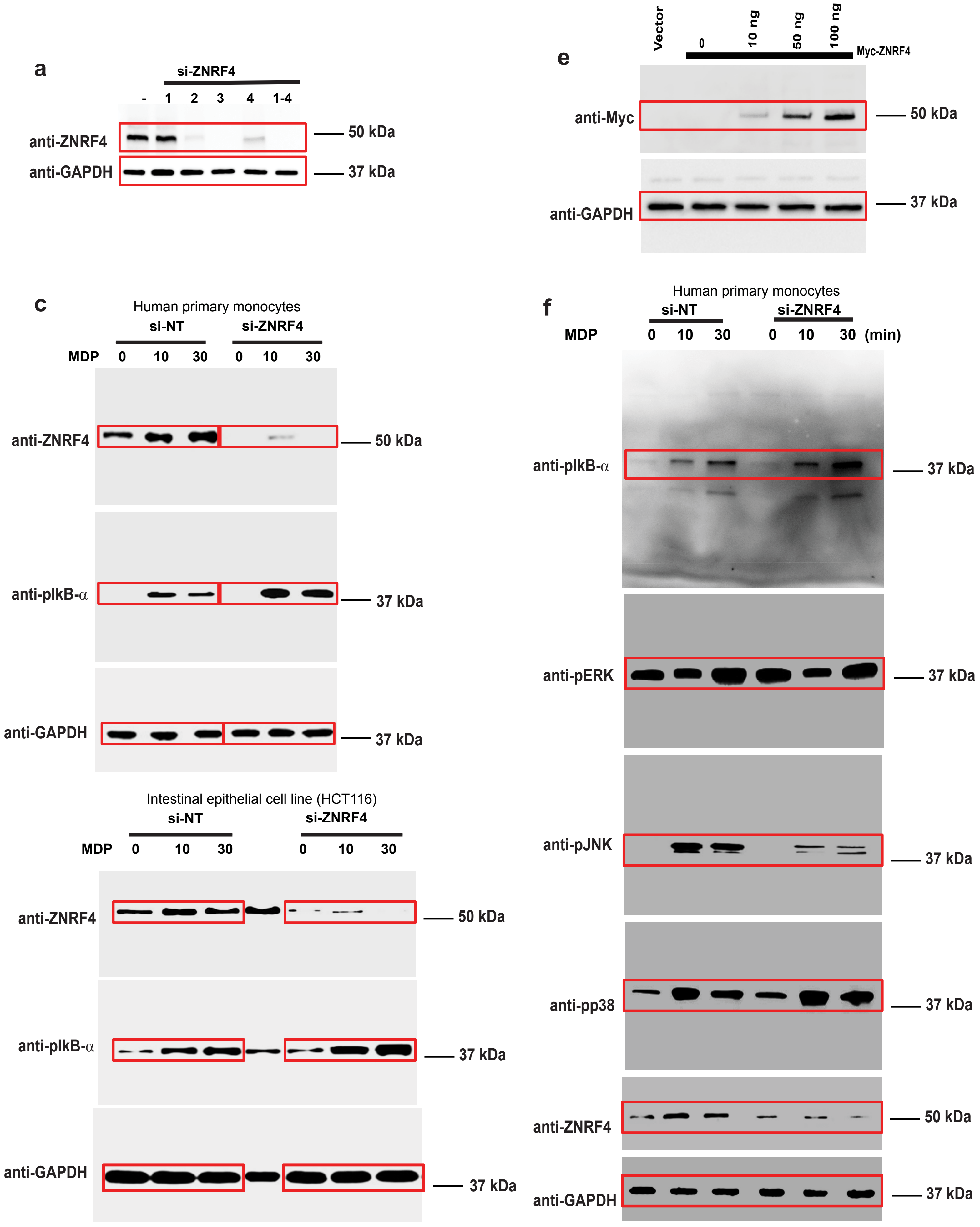

Supplementary Figure 6: Full length Western blots. Uncropped images of the blots probed for the indicated proteins as presented in Fig. 2.

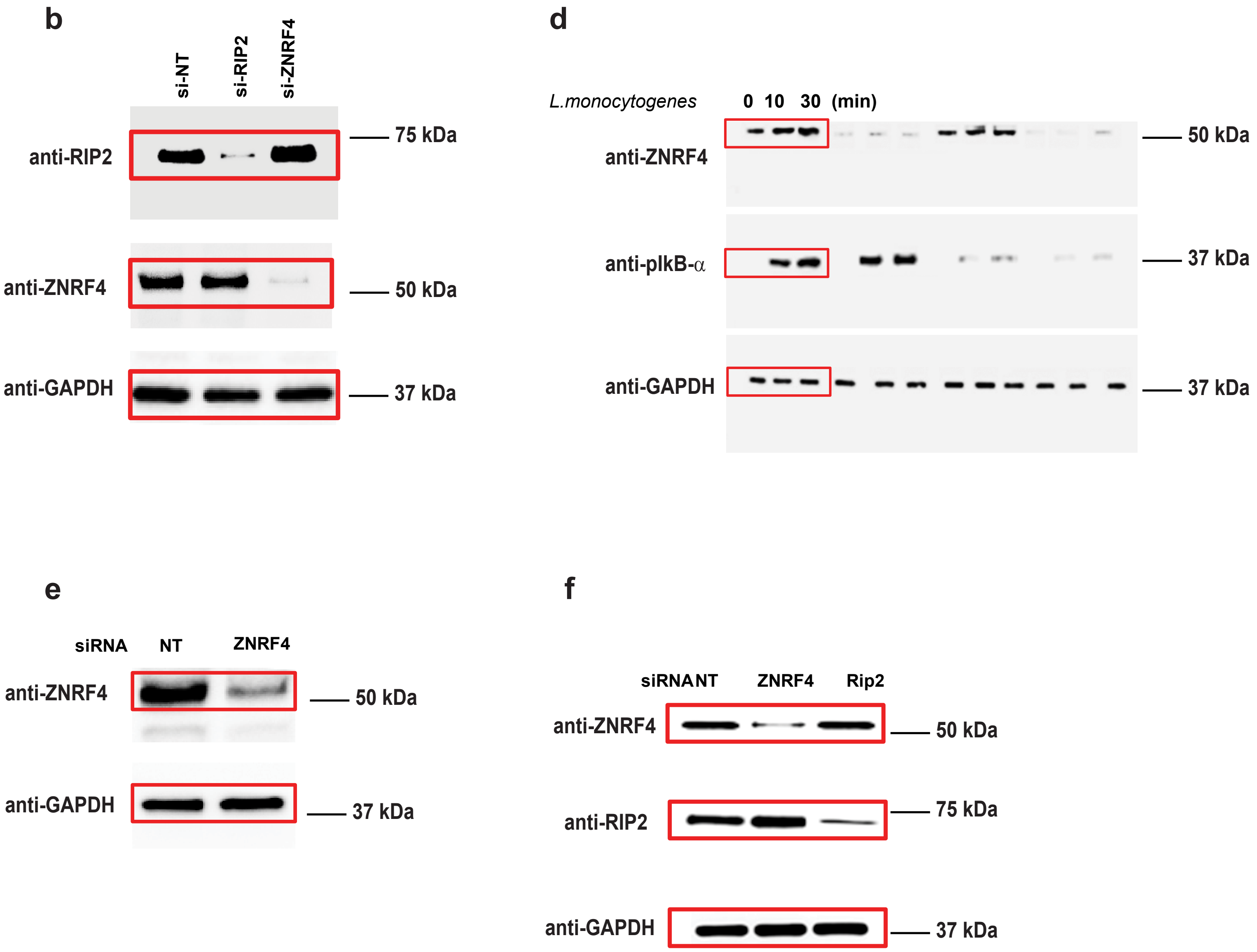

Supplementary Figure 7: Full length Western blots. Uncropped images of the blots probed for the indicated proteins as presented in Fig. 3.

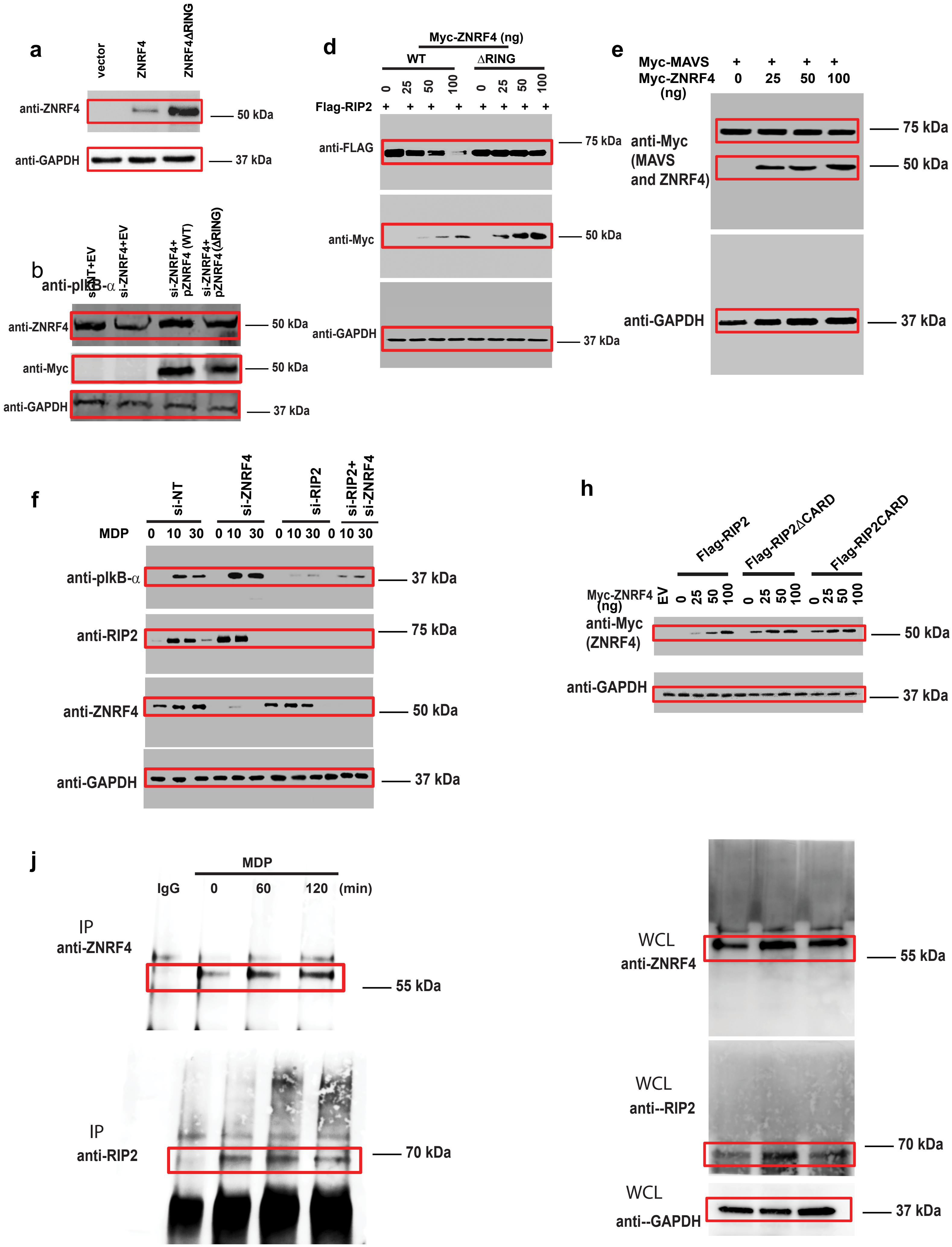

Supplementary Figure 8: Full length Western blots. Uncropped images of the blots probed for the indicated proteins as presented in Fig. 4.

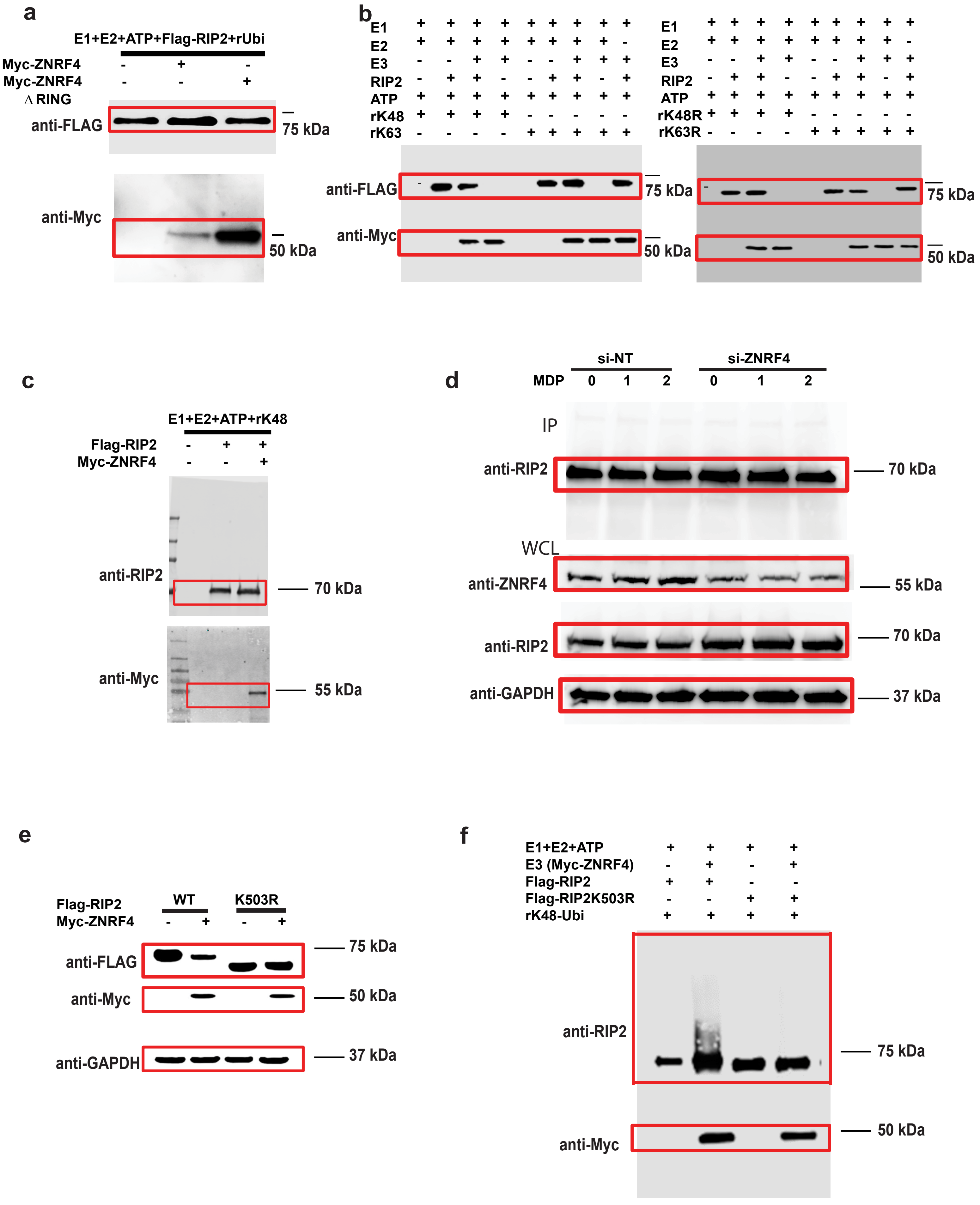

Supplementary Figure 9: Full length Western blots. Uncropped images of the blots probed for the indicated proteins as presented in Fig. 5.

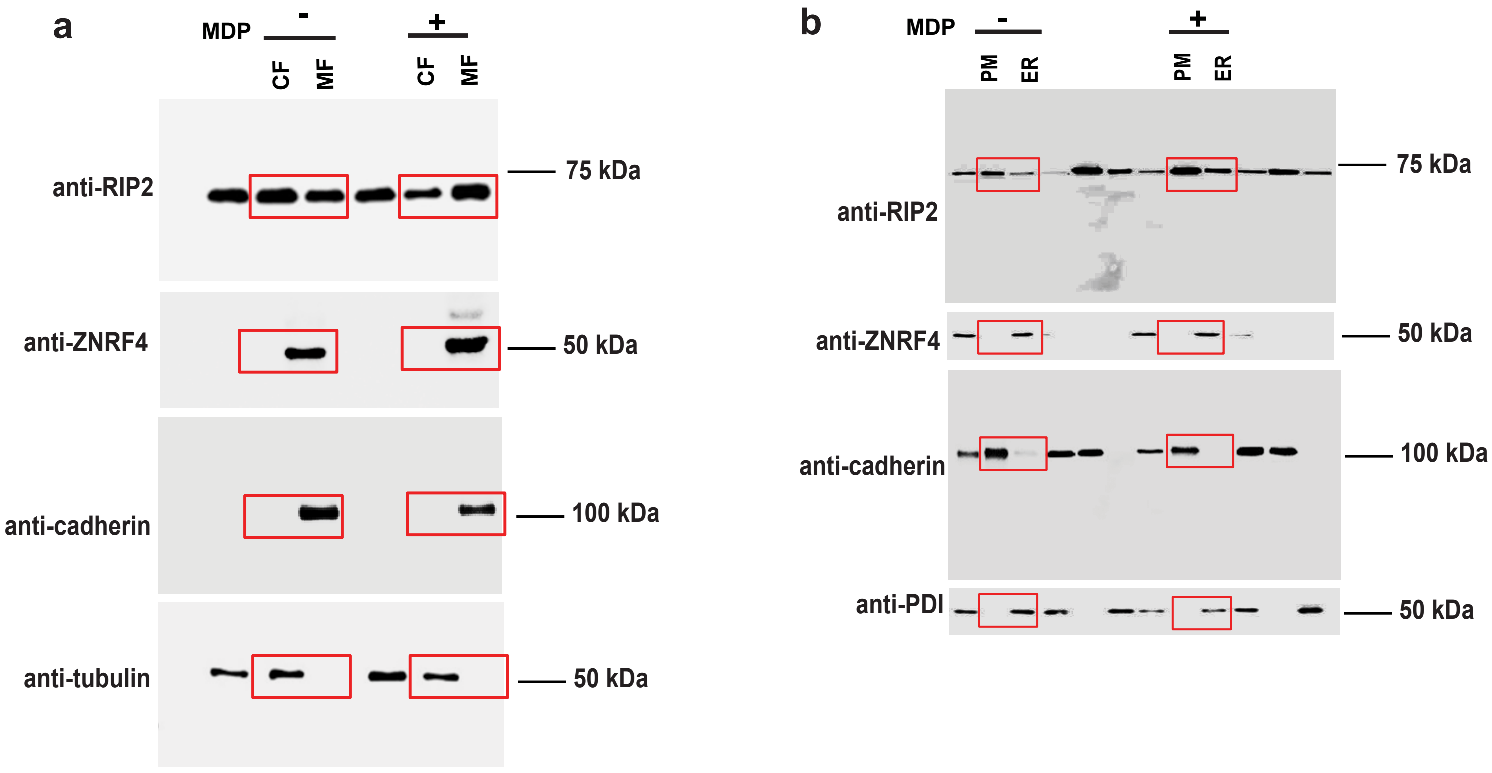

Supplementary Figure 10: Full length Western blots. Uncropped images of the blots probed for the indicated proteins as presented in Fig. 6.

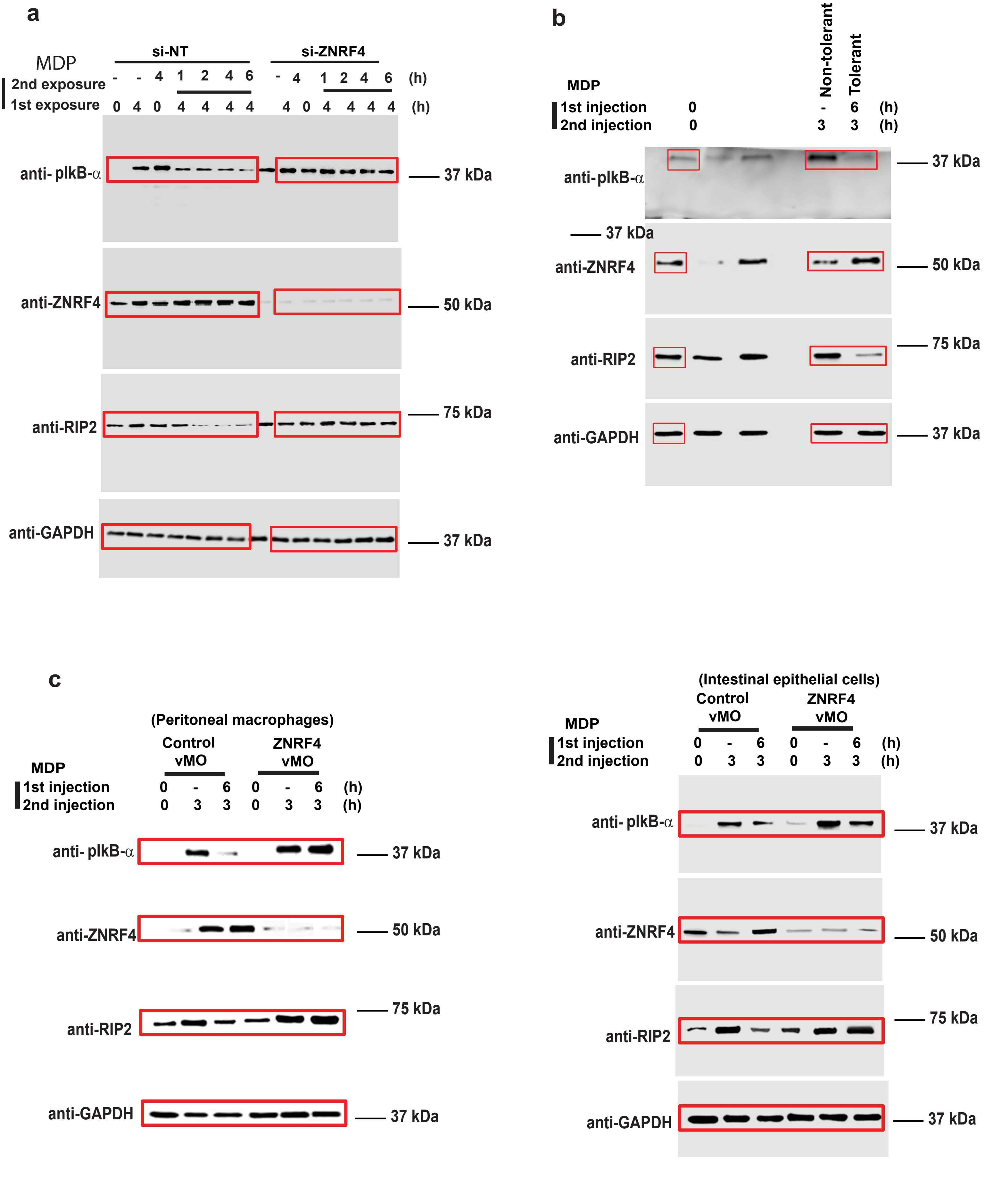

Supplementary Figure 11: Full length Western blots. Uncropped images of the blots probed for the indicated proteins as presented in Fig. 7.

Supplementary Table 1: Sequences of siRNAs and primers used

Sequence of siRNAs used

ZNRF4-si2	5’-CCUCAGUGUUCGUGAGCGA-3’
ZNRF4-si3	5’-CCGACGACCUCGUGAGCAU-3’
ZNRF4-si4	5’-GUAUGAGGAGGGCGACCAA-3’
RIP2-si1	5’-GGAAUUAUCUCUGAACAUA-3’
RIP2-si2	5’-GAAAGGAUGUCUUAAGAGA-3’
NT-si	5’-UAAGGCUAUGAAGAGAUACUU-3’
ZNRF4-3'si1	5'-GAAUAAAGUGGGUUUGAAAUU-3'
ZNRF4-3'si2	5'-GGUGCAAUGAGGAAUGUUUUU-3'
ZNRF4-3'si3	5'-AGAAUAAAGUGGGUUUGAAUU-3'

Sequence of primers used

ZNRF4-FULLForward	5'-GTGAGAATTCGGCCGCTCTGCCGTCCGGAGCACTT-3’
ZNRF4-FULLReverse	5'-GTCAGGTACCTTACTGACCAGGGGCCTCAGGA-3'
ZNRF4-QPCR-Forward	5’ AGTCAAAGCCTTGCTGGTCT 3’
ZNRF4-QPCR-Reverse	5’ CTGGCTTGACCTCCATCAG 3’
RIP2-Forward	5′-CCATCCCGTACCACAAGCTC-3′
RIP2-Reverse	5′-GCAGGATGCGGAATCTCAAT-3′
human β actin-Forward	5′-TGATATCGCCGCGCTCGTCGTC-3′
human β actin-Forward	5′-GCCGATCCACACGGAGTACT-3′
mouse *Il-6*-forward primer	5′-CCGGAGAGGAGACTTCACAG-3′
mouse *Il-6*-reverse primer	5′-TCCACGATTTCCCAGAGAAC-3′
mouse *gapdh*-forward primer	5′-CAAGGAGTAAGAAACCCTGGACC-3′
mouse *gapdh*-reverse primer	5′-CGAGTTGGATAGGGCCTCT-3′
